# Metabolic Signatures Elucidate the Effect of Body Mass Index on Type 2 Diabetes

**DOI:** 10.3390/metabo13020227

**Published:** 2023-02-03

**Authors:** Qiuling Dong, Sidra Sidra, Christian Gieger, Rui Wang-Sattler, Wolfgang Rathmann, Cornelia Prehn, Jerzy Adamski, Wolfgang Koenig, Annette Peters, Harald Grallert, Sapna Sharma

**Affiliations:** 1 Research Unit of Molecular Epidemiology, Helmholtz Zentrum München, 85764 Neuherberg, Germany; 2 Institute of Epidemiology, Helmholtz Zentrum München, 85764 Neuherberg, Germany; 3 Faculty of Medicine, Ludwig-Maximilians-University München, 81377 Munich, Germany; 4 Institute for Medical Information Processing, Biometry and Epidemiology (IBE), Ludwig-Maximilians-Universität München, 81377 Munich, Germany; 5 German Center for Diabetes Research (DZD), 85764 München-Neuherberg, Germany; 6 Institute of Translational Genomics, Helmholtz Zentrum München, 85764 Neuherberg, Germany; 7 Institute for Biometrics and Epidemiology, German Diabetes Center, Leibniz Center for Diabetes Research, Heinrich Heine University Düsseldorf, 40225 Düsseldorf, Germany; 8 Metabolomics and Proteomics Core Facility, Helmholtz Zentrum München, 85764 Neuherberg, Germany; 9 Institute of Experimental Genetics, Helmholtz Zentrum München, German Research Center for Environmental Health, Ingolstädter Landstraße 1, 85764 Neuherberg, Germany; 10 Department of Biochemistry, Yong Loo Lin School of Medicine, National University of Singapore, 8 Medical Drive, Singapore 117597, Singapore; 11 Institute of Biochemistry, Faculty of Medicine, University of Ljubljana, Vrazov trg 2, 1000 Ljubljana, Slovenia; 12 German Research Center for Cardiovascular Disease (DZHK), Partner site Munich Heart Alliance, 81377 Munich, Germany; 13 Deutsches Herzzentrum München, Technische Universität München, 81377 Munich, Germany; 14 Institute of Epidemiology and Medical Biometry, University of Ulm, 89069 Ulm, Germany; 15 Chair of Epidemiology, Faculty of Medicine, Ludwig-Maximilians-University München, 81377 Munich, Germany; 16 Chair of Food Chemistry and Molecular Sensory Science, Technical University of Munich, 85354 Freising-Weihenstephan, Germany

**Keywords:** obesity, type 2 diabetes, metabolomics, mediation, mendelian randomization, type 2 diabetes pathology

## Abstract

Obesity plays an important role in the development of insulin resistance and diabetes, but the molecular mechanism that links obesity and diabetes is still not completely understood. Here, we used 146 targeted metabolomic profiles from the German KORA FF4 cohort consisting of 1715 participants and associated them with obesity and type 2 diabetes. In the basic model, 83 and 51 metabolites were significantly associated with body mass index (BMI) and T2D, respectively. Those metabolites are branched-chain amino acids, acylcarnitines, lysophospholipids, or phosphatidylcholines. In the full model, 42 and 3 metabolites were significantly associated with BMI and T2D, respectively, and replicate findings in the previous studies. Sobel mediation testing suggests that the effect of BMI on T2D might be mediated via lipids such as sphingomyelin (SM) C16:1, SM C18:1 and diacylphosphatidylcholine (PC aa) C38:3. Moreover, mendelian randomization suggests a causal relationship that BMI causes the change of SM C16:1 and PC aa C38:3, and the change of SM C16:1, SM C18:1, and PC aa C38:3 contribute to T2D incident. Biological pathway analysis in combination with genetics and mice experiments indicate that downregulation of sphingolipid or upregulation of phosphatidylcholine metabolism is a causal factor in early-stage T2D pathophysiology. Our findings indicate that metabolites like SM C16:1, SM C18:1, and PC aa C38:3 mediate the effect of BMI on T2D and elucidate their role in obesity related T2D pathologies.

## 1. Introduction

According to the World health Organization (WHO), over 1 billion people worldwide are obese, including 650 million adults, 340 million adolescents and 39 million children, and this results in the degradation of health [1]. Obesity is a disease impacting most body systems and contributes to a range of noncommunicable diseases including cardiovascular disease, type 2 diabetes (T2D), and cancer [2,3,4]. It has been proven that being overweight or obese are the most critical conditions for risk of developing T2D and both are linked to metabolic syndrome [5]. Metabolic processes are regulated by various perturbations from its surrounding environment and several levels of enzymes [6]. The molecular mechanisms by which obesity affects T2D development include lipid metabolism, insulin sensitivity, and inflammation [7].

Increasing interest has been addressed in the application of metabolic profiling to the identification of disease biomarkers, as it is a potent approach to uncovering the convoluted progression between obesity, metabolism, and diabetes [8]. Stevens et al. outlined the metabolomic signature of human obesity and linked them to T2D parameters such as C-reactive protein (CRP) and HbA1c [9]. The study by Tulipani et al. shows metabolic traits [lyso]glycerophospholipids in particular lysophosphatidylcholines associated with morbid obesity and several amino acids glutamate, glycine and branch chain amino acids were biomarkers of risk of diabetes onset associated with obesity and prediabetes [10]. Lipidomics analysis has unraveled that several sphingomyelins, diacyl phosphatidylcholine, and lysophosphatidylcholine were associated with waist circumference whereas HOMA-IR was strongly related with specific lysophosphatidylcholines and diacyl phosphatidylcholines [11]. These studies provide support for the involvement of metabolites in progression of metabolic disease, but no emphasis was given to dissect the intermediate pathway between obesity and diabetes.

Small molecular lipids such as sphingolipids, glycerophospholipids, and fatty acids play vital roles in metabolic pathways related to health and disease. Sphingolipids are a class of lipids; simple sphingolipids include the sphingoid bases and ceramides. Ceramides are important bioactive lipids produced from three pathways: (i) the de novo pathway; (ii) the sphingomyelin pathway; and (iii) the salvage/recycling pathway [12]. Glycerophospholipids are a class of lipids that constitute a major component of cell membrane, which is generally composed of hydrophobic fatty acids and a hydrophilic phosphate group. The phosphate group is modified by different small molecules to form different kinds of glycerophospholipids, for example, by choline to form phosphatidylcholine [13]. Clinical studies have demonstrated that phospholipids including sphingolipids and glycerophospholipids are strongly associated with insulin sensitivity [14].

Genetic composition can be used to make predictions regarding disease susceptibility. The overgrown obesity rates and their clinical consequences (T2D) clearly indicate that non-genetic or environmental factors and their interaction with genetic variants are major players of disease development [15]. Genome-wide association studies show more than 900 genetic variants associated with BMI [16] and more than 230 loci influencing risk of T2D [17]. Furthermore, linking metabolites with other omics, especially genetics using genome-wide association (mGWAS), gives access to genetics’ influence on the metabolic composition of key lipids, amino acids, and carbohydrates [18,19,20]. mGWAS, with a growing sample size and ascending complex metabolic traits, allows for a more comprehensive and systems-based downstream analysis.

In this work, we considered a targeted metabolomic analysis of 1715 participants enrolled in the KORA FF4 Cohort to investigate metabolite markers for obesity and T2D participate in development of obesity-related Type 2 diabetes. Metabolite profiles of 146 named serum metabolites were assessed and compared with publicly available studies. The metabolites mediation effect of BMI on T2D was investigated using a mediation test. Further, we used mendelian randomization (MR) to define metabolites that may be causally linked with BMI and T2D and vice versa using genetic variants. Finally, biological pathways and consequences were analyzed by incorporating genetics and mouse model data from the literature, yielding the bioactive role of sphingolipids and glycerophospholipids in metabolic dysregulation and beta cell dysfunction.

## 2. Materials and Methods

### 2.1. Study Subjects and Sampling

The Cooperative Health Research in the Region of Augsburg (KORA) study is a population-based cohort study. The KORA FF4 study (2013–2014) is the second follow-up of KORA S4 (1999–2001). All samples included in the study were collected in the morning between 8:00 a.m. and 10:30 a.m. after at least 8 h of fasting. We examined 2216 individuals who had phenotype and metabolite measurements and excluded 501 participants in the analysis, including (1) underweight (BMI < 15 kg/m^2^) or missing covariate values (*n* = 23), and (2) prediabetes (impaired fasting glycemia or impaired glucose tolerance, *n* = 390). It is reported that impaired fasting glucose and impaired glucose tolerance should be considered as different phenotypes from T2D, so we removed these participants [21]. Additionally excluded were (3) diagnosis for type 1 diabetes (*n* = 6) and (4) unclear type of diabetes mellitus (*n* = 82). The remaining dataset has 1715 participants, comprising 1276 non-obese participants (BMI < 30 kg/m^2^) and 439 obese (BMI ≥ 30 kg/m^2^), and 1415 non-diabetic participants and 300 individuals with type 2 diabetes. The incident T2D was defined based on an oral glucose tolerance test (OGTT) or a validated physician diagnosis. WHO diagnostic criteria were applied to the classification of KORA participants.

### 2.2. Metabolite Quantification and Normalization

Samples were collected and stored at −80 °C and profiling FF4 metabolomics were performed in February–October 2019. The stability was measured and validated [22]. Blood samples from KORA FF4 participants in the study were measured with the Absolute*IDQ*^TM^ p180 Kit (BIOCRATES Life Sciences AG, Innsbruck, Austria). The assay procedures were previously described in detail [23]. Briefly, 10 μL serum samples were added to the 96-well kit plate with respective standards and dried under a nitrogen stream. Amino acids and biogenic amines were derivatized with 5% phenylisothiocyanate in ethanol/water/pyridine. After metabolite and standard extraction, using methanol containing 5 mM ammonium acetate, the eluate was diluted with water for LC MS/MS analysis and with the kits running solvent for FIA-MS/MS analysis. The analytical process was conducted by the MetIQ™ software package and a targeted profiling scheme was applied to quantitatively identify known metabolites. Metabolites that met any one of the three exclusion criteria were deleted: (1) coefficient of variance (CV) value of five reference samples was equal to or greater than 25%; (2) there were ≥ 50% of all measured sample concentrations lower than corresponding plate limit of detection (LOD), the plate LOD was defined as 3 times median of three zero samples in each plate; and (3) the non-detectable rate of all measured samples was equal to or greater than 50%. There were 146 metabolites that passed quality control (QC). Non-detectable values in sample data were randomly imputed ranging from 75% to 125% of the half of the lowest measured value of the metabolite in each plate. Afterwards, plate normalization factors (NFs) were taken into consideration and adjusted for metabolite concentrations to reduce the plate impact. The normalization process was described elsewhere [24]. Metabolite concentrations were natural-log transformed and scaled (mean = 0, sd  =  1) to ensure comparability between the metabolites.2.3. Statistics

All statistical analyses were performed in R (version 4.1.0) and a two-sided *p* value < 0.05 was considered as statistically significant after the Bonferroni correction.

#### 2.2.1. Multivariable Linear Regression and Logistic Regression

For BMI-metabolite associations, multivariable linear regression was employed with each metabolite as an independent variable and the BMI value as a dependent variable. This analysis was adjusted for covariates age, sex in basic model and including additional covariates like, physical activities, smoking status, systolic blood pressure, high-density lipoprotein cholesterol (HDL-C), triglyceride, fasting glucose levels in full model. In logistic regression analysis for metabolite-T2D associations, odds ratios (ORs) for each metabolite between two groups were calculated. Logistic regression analysis was carried out with the diabetic status as a dependent variable and each metabolite as an independent variable. Same risk factors in the linear regression analyses with additional BMI were added as covariates in the logistic regression model and the same significance level was adopted.

#### 2.2.2. Sobel Mediation Test

We performed Sobel tests [25,26] to assess whether metabolites carry the influence of BMI to T2D. All analyses were conducted in R by using the package ‘bda’ v15.2.5 and the functions mediation test. In order to adjust confounders, the residuals were obtained from a linear regression model that each metabolite was a dependent variable and covariates (age, sex, physical activity, smoking status, systolic blood pressure, HDL-C, and triglyceride) as independent variables. Afterwards, metabolite residual entered the Sobel test model as a mediator, and BMI as an independent variable, whereas fasting glucose or HbA1c was taken as the dependent variable. With these two approaches, we examined the mediation effect of metabolites. The *p*-value thresholds follow the Bonferroni-correction and metabolites with *p* < 0.05 were considered to have a significant mediation effect.

#### 2.2.3. Mendelian Randomization

We checked for causal inference using two sample mendelian randomisation (2SMR) methods from the MRInstruments (0.3.2) and TwoSampleMR library (v0.5.6) [27]. 2SMR is a method to draw a causal relation using only summary statistics of genome wide association studies (GWAS) from two observational studies [27]. To assess the impact of BMI on metabolite levels, in a 2SMR test, BMI instruments were obtained from the GIANT-UK Biobank meta-analysis [16] and the corresponding SNP estimates on T2D were extracted from the mGWAS [28]. BMI instruments with genome-wide significance (*p* < 1 × 10^−8^) and an LD clumping threshold of 0.001 were considered. The exposure and outcome data were harmonized before performing the MR analysis by positioning the SNPs on the same effect allele. We used the IVW method to estimate the causal effect of BMI on metabolites. From the direction of metabolites to T2D, metabolite instruments were obtained from the metabolite-GWAS [28] and extracted the corresponding SNPs from the GWAS meta-analysis [29]. After LD clumping and harmonization, a Wald ratio method was selected in MR analysis to estimate the causal relationship due to the limited SNP instruments. For sensitivity analysis, we performed heterogeneity or horizontal pleiotropy based on the MR-Egger analysis.

## 3. Results

### 3.1. Associations of Metabolites with BMI and T2D

#### 3.1.1. Characteristics of the KORA FF4 Participants

Among 1715 participants, 1276 individuals were non-obese (BMI < 30) and 439 were obese (BMI ≥ 30). As shown in Table 1, there was no significant difference in sex and alcohol consumption between obese and non-obese groups. Compared with the non-obese group, the blood pressure, triglycerides, and fasting glucose were significantly higher and HDL cholesterol was significantly lower in the obese group. Besides, for participants with BMI < 30, only 136 individuals (10.7%) developed T2D, whereas T2D was diagnosed more frequently in obese participants (37.6%).

Similarly, for alcohol consumption, no significant difference between healthy and T2D participants was observed. BMI, blood pressure, triglycerides, and fasting glucose were significantly higher and HDL cholesterol was significantly lower in the T2D group (Table 2). Compared with non-diabetic individuals, the cases of obesity in T2D groups (53.3%) were almost three times higher than in the normal participant’s group (19.2%).

#### 3.1.2. Metabolites Associated with BMI and T2D

A linear regression model was used to investigate the BMI associated metabolites and a logistic regression model was employed for T2D associations. Model assumptions have been performed and reported in Supplemental Document S2. Only age and sex (adding BMI for T2D model) were added in the basic regression models. The numbers of significant metabolites were the highest, and 83 metabolites were significantly associated with BMI and 51 metabolites were significantly associated with T2D.

**Figure 1:**
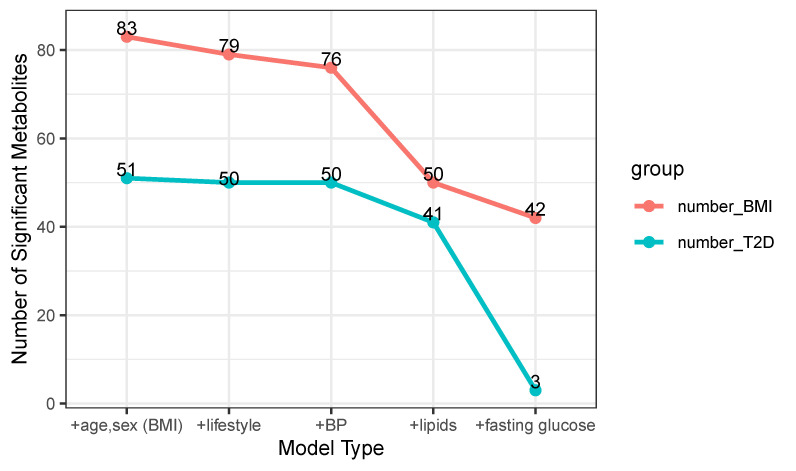
The number of metabolites significantly associated with BMI and T2D in different models after multiple testing correction. The first coordinate on *x*-axis shows basic model building upwards with including lifestyle, blood pressure, lipids, and fasting glucose parameters as covariates in the model. The *y*-axis depicts a number of significant metabolites resulting from each model as indicated on *x*-axis. Lifestyle includes smoking status and physical activities. BP: systolic blood pressure; lipids include HDL cholesterol (HDL-C) and triglycerides.

Next, we tested how covariates like lifestyle, lipids, and fasting glucose influenced the association between metabolites with BMI and T2D. When more covariates were included, the significant numbers decreased. In particular, the association between BMI and metabolites was affected mostly by lipids and blood pressure, which was indicated from the dramatically dropped number when lipids and blood pressure were added in the model. Fasting glucose influenced mostly the T2D association and the number of significant metabolites decreased from 41 to 3, which suggests many metabolites were associated with T2D mediated by fasting glucose. (Figure 1)

**Figure 2:**
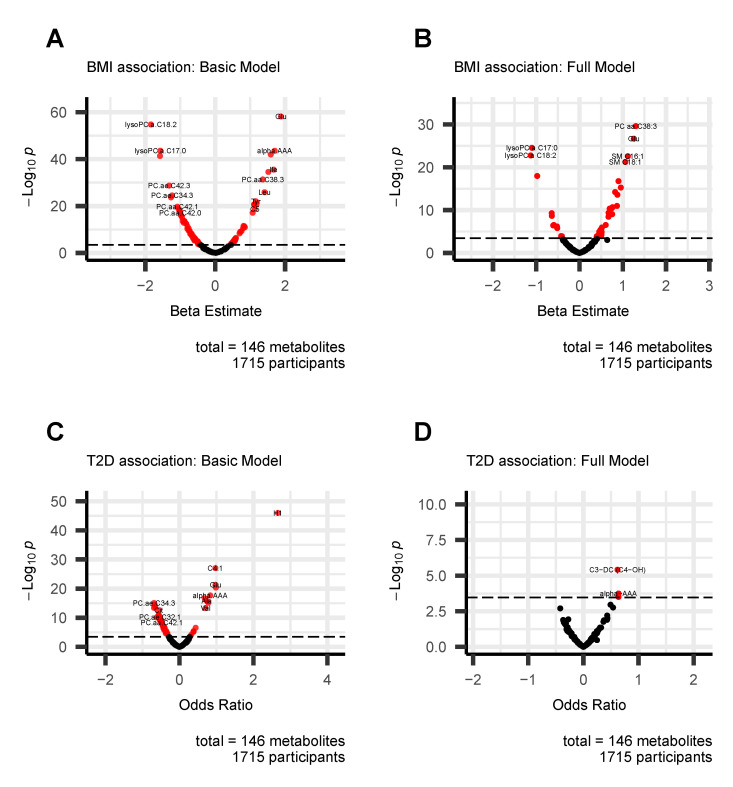
Volcano plots show the association of metabolites with BMI and T2D in the basic model (**A**,**C**) and the full model (**B**,**D**). Bonferroni correction *p*-value cut-off is 0.05/146 = 0.00034 was considered. Each dot represents a metabolite, and they are displayed based on the beta estimate or odds ratio (*x*-axis) and the negative logarithm (base 10) of the *p*-value (*y*-axis). The covariates for the basic model are age, sex, and (BMI); the covariates for the full model are age, sex, (BMI), smoking status, physical activities, HDL-C, blood pressure, triglycerides, and fasting glucose.

Obesity specific metabolites: Linear regression was used to execute a metabolite-wide association study in KORA FF4, and we identified 83 and 42 metabolites associations in the basic and full models after conservative Bonferroni correction for multiple testing. A volcano plot (Figure 2A,B) provides a quick visual identification of statistically significant metabolites with a larger effect size. The full summary statistics of different models are reported in the Supplemental Materials Tables S2 and S3. Table 3 shows only the metabolites significantly associated with BMI in the full model. Totally, 12 metabolites were negatively associated with BMI whereas 30 were positively associated in the full model. We confirmed the BMI metabolites associations using the published literature and almost all were replicated except for SM C20:2.

From this analysis we made the following four key observations.

(1) We have observed that all diacyl phosphatidylcholines (PC aa), acylcarnitines, biogenic amines, and sphingomyelins (SM) were positively associated with BMI. In particular, PC aa C38:3 was the strongest metabolite associated with BMI (1.301 [1.082–1.520], q-value = 3.65 × 10^−28^. Glutamate (1.255 [1.032–1.478], q-value = 3.05 × 10^−25^), SM C16:1 (1.118 [0.901–1.336], q-value = 3.87 × 10^−21^), alpha-AAA (0.955 [0.726–1.184], q-value = 8.04 × 10^−14^), and C0 (0.672 [0.462–0.882], q-value = 6.13 × 10^−8^) were those with the strongest association in each category;
(2) Some amino acids were positively correlated with BMI. Among them, glutamate (1.255 [1.032–1.478], q-value = 3.05 × 10^−25^) and Tyrosine (0.901 [0.695–1.106], q-value = 2.51 × 10^−15^) have the strongest association. Others were inversely associated with BMI: Asparagine (−0.642 [−0.843–−0.44], q-value = 7.73 × 10^−8^) and Glycine (−0.515 [−0.724–−0.305], q-value = 2.34 × 10^−4^);
(3) Three acylalkylphosphatidylcholine (PC ae) were positively associated with BMI, PC ae C36:5 (0.502 [0.29–0.713], q-value = 5.09 × 10^−4^), PC ae C36:4 (0.457 [0.254–0.66], q-value = 1.56 × 10^−3^), and PC ae C32:2 (0.506 [0.258–0.754], q-value = 9.52 × 10^−3^); whereas others PC aes were negatively associated with BMI: PC ae C42:3 (−0.594 [−0.821–−0.368], q-value = 4.29 × 10^−5^), PC ae C36:2 (−0.607 [−0.84–−0.373], q-value = 5.48 × 10^−5^), PC ae C40:6 (−0.424 [−0.639–−0.209], q-value = 1.66 × 10^−2^), and PC ae C38:2 (−0.406 [−0.613–−0.199], q-value = 1.80 × 10^−2^);
(4) All lysophosphatidylcholines (lyso PC) were negatively associated with BMI. In particular, lysoPC a C17:0 (−1.1 [−1.305-−0.896], q-value = 4.20 × 10^−23^) was the strongest.

**Figure 3:**
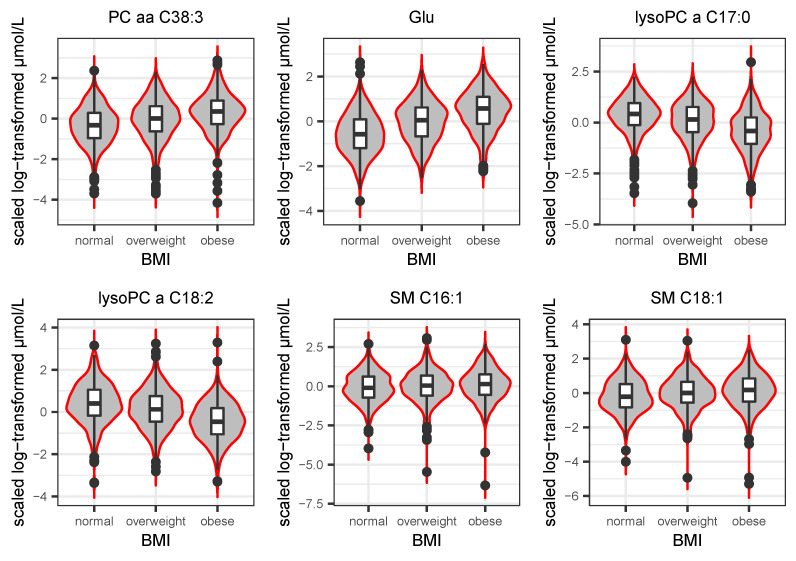
Violin-boxplots show the top six significant metabolite distributions of study subjects divided in three different classes of BMI, normal (18.5 ≤ BMI < 25), overweight (25 ≤ BMI < 30), and obese (BMI ≥ 30). The box contains 50% of the participants. The middle line stands for median dividing the box into two areas. The 25th and 75th percentile of the distribution are represented by upper and lower hinges.

To investigate the direction of effect across BMI class (normal, overweight, and obese), the six most significant metabolites from the full model were visualized by violin-box plots stratified by BMI in Figure 3. PC aa C38:3, glutameta (Glu), SM C16:1 and SM C18:1 showed synchronized direction with BMI, increasing concentrations with increased BMI, whereas lysoPC a C17:0 and lysoPC a C18:2 reversed, which is consistent with the result from the linear regression model.

T2D specific metabolites: multivariable logistic regression analysis was conducted with known diabetes-related variables as covariates to identify significant metabolites. Similarly, alcohol was not included in the model as a covariate because there was no significant difference between T2D and healthy individuals. A volcano plot (Figure 2C,D) represents the result of the logistic regression model. The full summary statistics of different models are reported in the Supplemental Materials Tables S4 and S5. Table 4 shows only the metabolites significantly associated with T2D in the full model. Three metabolites, C3-DC (C4-OH), alpha-AAA and isoleucine (Ile) were observed to have significant associations in the full model after conservative Bonferroni correction. All of them were positively correlated with T2D and replicated by the published literature (details in the Section 4).

**Figure 4:**
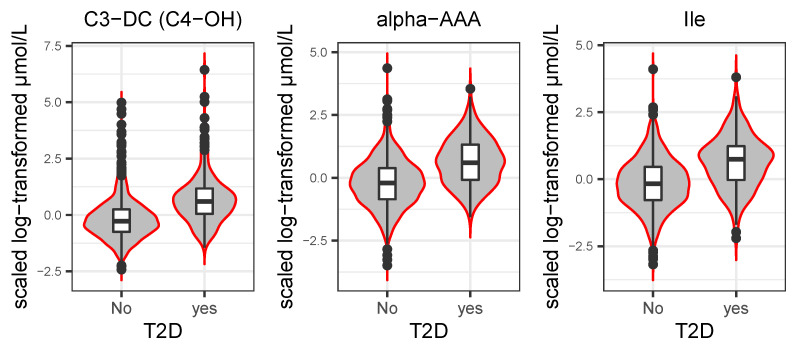
Violin-box plots show the distribution of three significant metabolites stratified by diabetic status. The box contains 50% of the observations. The middle line stands for median dividing the box into two areas. The 25th and 75th percentile of the distribution are represented by upper and lower hinges.

Figure 4 displays the violin-boxplots of the three significant metabolites in the T2D full model. The concentrations of C3-DC (C4-OH), alpha-AAA, and Ile increased among the group with T2D, which is consistent with the result from the logistic regression model.

### 3.2. Sobel Mediation Test

**Figure 5:**
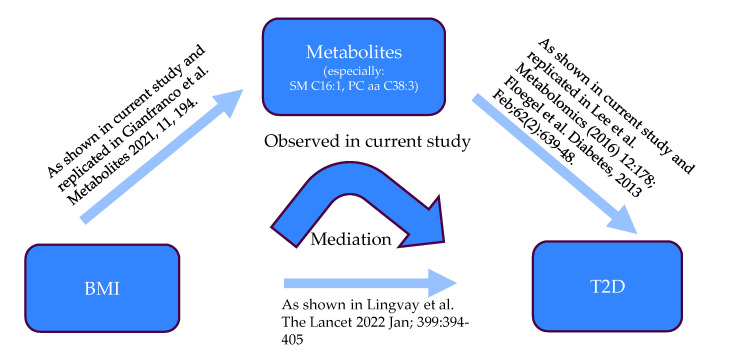
Schematic representation of the mediation analysis [2,30,31,32].

A Sobel mediation test was conducted to investigate whether a mediator carries the effect of an independent variable on a dependent variable. In our research, we used fasting glucose or HbA1c as T2D indicators to test the metabolite mediation of the effect of BMI on T2D. In order to adjust the influence of the confounders, the metabolite residual, calculated from the linear regression model between each metabolite and covariates, was used as a mediator in the test. The significant mediators are shown in Table 5 and full statistics are shown in Supplementary Materials Table S6 and Table S7, respectively. The mediation of the associations between BMI and fasting glucose via the 12 metabolites were Bonferroni-corrected significant (q-value < 0.05) whereas nine metabolite mediations were significant between BMI and HbA1c. Among all these metabolites, sum of hexose, SM C16:1, glutamate, PC aa C38:3, alpha-AAA, isoleucine, lyso PC a C18:0, and leucine were significant in both tests, which suggests their robust mediation effects. The sum of hexose owned the strongest mediation in both studies, which was not very surprising as it mainly represents the glucose in human blood. A summarizing plot of the mediation analysis is shown in Figure 5.

### 3.3. Mendelian Randomization

**Figure 6:**
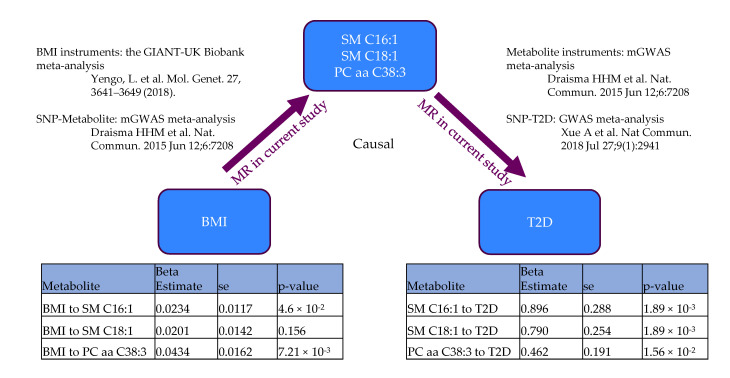
Schematic diagram is suggestive of relationships between BMI, metabolites and T2D. The studies we used for MR were listed in the figure. ß-estimate stands for beta coefficient, se stands for standard error [16,28,29].

To assess the causality relationship between BMI, identified metabolites from mediation test and T2D, we employed two-sample mendelian randomization (MR) tests. We conducted a two-sample (2SMR) mendelian randomization analysis in two directions (BMI-to-metabolite, metabolite-to-T2D, Figure 6). BMI instruments were extracted from the GIANT-UK Biobank meta-analysis [16] and then the corresponding SNPs estimated on T2D were selected from the published metabolite-GWAS [28]. Metabolite instruments were obtained from the same metabolite-GWAS [28] and extracted the corresponding SNPs from the GWAS meta-analysis [29]. The 2SMR analysis results are presented using the Inverse Variance Weighted (IVW) method in BMI to metabolite direction and the Wald ratio method in metabolite to T2D direction. Only SM C16:1, SM C18:1, and PC aa C38:3 have available instruments in both directions, so we showed the MR results of these three metabolites in this study.

Our results indicated that the change of BMI could cause the concentration change of SM C16:1 and PC aa C38:3. The change of SM C16:1, SM C18:1, and PC aa C38:3 contributes to the development of T2D, which suggests lipids like SM C16:1 and PC aa C38:3 are intermediate molecules involved in the progression from obesity to T2D. Sensitivity analysis was carried out to test if these results were robust from proof of heterogeneity or horizontal pleiotropy, which was supported by the MR-Egger analysis. For BMI to SM C16:1, Q statistic from the heterogeneity measure was not significant (p_Het 0.51 > 0.05), indicating there was no heterogeneity. For BMI to PC aa C38:3, the *p*-value (p_Het 0.03) was slightly lower than 0.05, showing heterogeneity between different instruments, and random effect was selected to report the result. The MR–Egger intercept test (p_Pleio > 0.05) suggested no directional pleiotropy for both metabolites. For the direction of metabolites to T2D, we did not perform the sensitivity analysis as only one SNP instrument was available for each metabolite.

### 3.4. The Biological Role of SM C16:1, SM C18:1, and PC aa C38:3 in Transition to T2D

**Figure 7:**
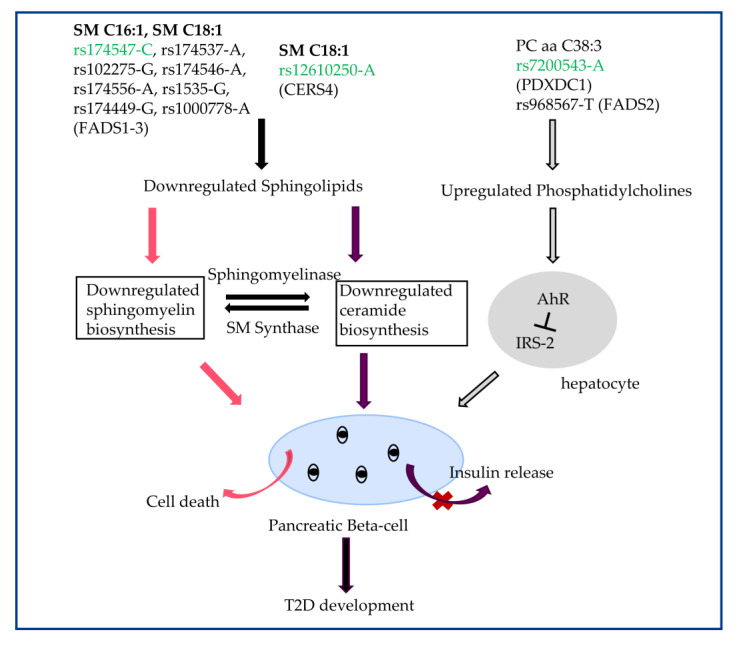
Schematic representation of the pathway analysis of diminished sphingolipid metabolism to a transition of T2D. The SNPs marked with green are the ones used in the MR test. The red pathway is generally involved in sphingomyelins (SM), the purple and gray pathways are for ceramides, and phosphatidylcholines (PC), respectively. All three kinds of metabolites influence insulin release.

In order to understand the biological pathway of these three lipids (SM C16:1, SM C18:1, and PC aa C38:3), we searched for the associated SNPs and genes in humans. The metabolite SM C18:1 was reported to be associated with SNP rs12610250-A, the locus CERS4 [28]. PC aa C38:3 was significantly correlated with rs7200543-A, locus PDXDC1 and rs968567-T, locus FADS2 [28]. Both SM C16:1 and SM C18:1 were associated with rs174547-C, rs174537-A, rs102275-G, rs174546-A, rs174556-A, rs1535-G, rs174449-G, rs1000778-A, the locus FADS1-3 [33]. CERS4 and FADS1-3 were identified to influence the biosynthesis of sphingolipids including sphingomyelins and ceramides [28,33], which could be produced from each other by hydrolysis and synthase [34]. It was reported sphingomyelins were essential for insulin secretion in rat beta cells [35] and beta cell viability [36]. Mice model and cell experiments demonstrated that inhibition of ceramide biosynthesis impaired insulin sensitivity and caused pancreatic beta-cell dysfunction [36,37]. This is consistent with the result of negative associations between SM C16:1, SM C18:1, and T2D in the current study (basic model). The specific variants of PDXDC1 and FADS2 were found to upregulate phosphatidylcholine [28]. Increased phosphatidylcholines bind to and activate the aryl hydrocarbon receptor (AhR) expressed in hepatocytes and inhibition of the essential genes including IRS-2 for promotion of the insulin pathway [38]. We observed the consistent result that PC aa C38:3 was positively associated with T2D in a human study [32]. These observations support the particular sphingolipid and phosphatidylcholine dysmetabolism as a causal factor in early-stage T2D progression (shown in Figure 7).

## 4. Discussion

Obesity triggers a cascade of metabolic processes that raise the stake of various comorbidities including insulin resistance and glycemic deterioration causing T2D. Understanding the role of intermediate molecules involved in the process from obesity to T2D offers a therapeutic strategy to early-stage T2D pathophysiology. In our study, we assessed the functionally characterized targeted metabolite profiles of KORA FF4 participants for underlying metabolic pathway links. The major results of the present study are (1) identification of several metabolite changes among subjects with obesity and diabetic status, (2) metabolites such as SM C16:1, SM C18:1, and PC aa C38:3 show significant mediation effect of BMI on T2D, (3) the causality direction of BMI, three lipids (SM C16:1, SM C18:1, PC aa C38:3), and T2D, and (4) the biological consequences of the downregulated sphingolipids and upregulated phosphatidylcholine.

It is strongly suggested that in blood, elevated concentrations of branched-chain amino acids are associated with an increased risk of type 2 diabetes mellitus [39,40]. In our study among metabolites associated with BMI, the branched chain amino acids (BCAAs), isoleucine (Ile), leucine (Leu), and valine (Val) were positively correlated and have been confirmed in several studies [30,41,42].In fact, isoleucine was positively associated with T2D in the full model and replicated in the literature [21]. Isoleucine (Ile) and leucine (Leu) also appear to be mediators between BMI and T2D. Other amino acids such as glutamate, alanine, tyrosine, and phenylalanine significantly changed among different BMIs and these also have been found in other studies [30,43,44]. Other studies speculate the reason could be that high concentration of BCAAs causes insulin resistance by activating the mammalian target of rapamycin (mTOR) signaling [45,46]. There might be a mechanism proposed for branched-chain-keto acid dehydrogenase (BCKD) inhibition and suppression of enzymatic catabolism of amino acids in individuals with obesity [47].

Acylcarnitines like carnitine (C0), valerylcarnitine (C5), propionylcarnitine (C3) increased in individuals with higher BMI, which is in line with other studies [30,44]. Hydroxybutyrylcarnitine (C3-DC (C4-OH)) was positively associated with T2D [48]. Several studies indicate an increase in plasma acyl carnitines in patients with T2D [30,31] and it is attributed to an incomplete long chain fatty acyl-CoA oxidation of fatty acids [43,49].

Biogenic amines were found to be related with obesity and T2D. Alpha-aminoadipic acid (alpha-AAA) and kynurenine were positively associated with BMI. Meantime, alpha-aminoadipic acid was also positively associated with T2D in the full model and showed significant mediation of BMI to T2D. Alpha-aminoadipic acid is an intermediate in the metabolism of lysine and rat studies indicate that aminoadipic acid is elevated in the pre-diabetic state and so it could be a predictive biomarker for the development of diabetes [50].

Considering glycerophospholipids, all diacylphosphatidylcholines (PC aa) increased with increased BMI, such as PC aa C38:3, PC aa C38:4, PC aa C40:4, PC aa C32:1, and especially PC aa C38:3, the strongest metabolite with the lowest *p*-value, which is in line with Frigerio et al. [30]. All lysophosphatidylcholines (lyso PCs) were observed to have negative association with BMI. lysoPC a C17:0, lysoPC C18:2, and lysoPC C18:1 were the strongest negatively correlated with BMI, consistent with several other studies [10,51]. Only a few acylalkylphosphatidylcholine (PC ae) increased with BMI (PC ae C36:5, PC ae C36:4, PC ae C32:2) whereas many decreased (PC ae C42:3, PC ae C36:2, PC ae C40:6, PC ae C38:2). Moreover, PC aa C38:3, LysoPC a C16:0, LysoPC a C17:0, and LysoPC a C18:0 were observed to mediate from BMI to T2D, and this is a novel finding in our study. Phospholipids such as phosphatidylcholines (PC) are the essential constituent of cellular membranes and are critical for cellular signal transduction [52]. The LysoPCs (16:0, 17:0, 18:0) negatively associated with T2D in the basic model in our cohort have been considered to be involved in pro-inflammatory and atherogenic [53], but their major role still needs to be elucidated. PC aa C38:3 is reported to be positively associated with incident T2D [32], and mediation analysis and mendelian randomization results indicate it could be the intermediate molecules involved in obesity-related T2D development. The mechanisms governing the PC-mediated association between obesity and T2D could be via fatty acid (FA) and insulin signaling pathways. High-fat diets, inducing overproduction of PC, result in obesity and diabetes in individuals [54,55]. It is stated that abnormally high PC lipids affect energy metabolism and insulin signaling [56,57]. Mice fed with high-fat diets show upregulation of exosomal phosphatidylcholine, which results in binding to the aryl hydrocarbon receptor (AhR) [38], a transcription factor expressed in hepatocytes to integrate dietary and metabolic processes, and thus inhibition of the insulin response.

The Frigerio et al. study [30] confirms that sphingomyelins (SM), SM C16:1, and SM C18:1 were significantly associated with BMI. In the mediation test, both SM C16:1 and SM C18:1 have significant mediation effects of BMI on fasting glucose. These two metabolites have been shown to be associated with BMI and T2D in other studies [30,31]. Integrating with mendelian randomization suggests the causality direction and sphingomyelins such as SM C16:1 could be the molecular mediators of obesity-to-T2D evolution. Sphingomyelins are one of the most abundant sphingolipids in bodily fluids and in tissues, which is a lipid class with both signaling and structural properties and was reported to be related to the development of major metabolic and cardiovascular diseases [58,59,60]. The metabolic link between obesity and diabetes could be induced by modulating inflammation via FA and proinflammatory cytokines. Increased bioavailability of free fatty acid (FFA) and proinflammatory cytokines are characterized in obese subjects; sphingolipid metabolism is affected through both substrate supply and regulation of the enzymes [61,62]. Through the use in vivo and vitro mice models, it is confirmed that saturated FAs stimulate toll-like receptor 4 (TLR-4), activating sphingomyelinase (SMase) and converting sphingomyelins to ceramide, which reduces sphingomyelins content and exerts an action of insulin resistance [63]. SMase is also observed to be activated by proinflammatory cytokines tumor necrosis factor-alpha (TNF-α), resulting in an increased ceramide production from C57BL/6J mice with the intraperitoneal administration of TNF-α [64]. These events can lead to pancreatic β-cell dysfunction and T2D development in obese subjects. A study by Kelli M Sas et al. [65] investigates the role of perturbed ceramide metabolism in diabetic kidney disease (DKD). Ceramides were measured in the plasma and kidney cortex of a C57BLKS db/db mouse model of DKD which revealed long-chain ceramides (C14:0, C16:0, C18:0, C20:0) and a glucosylceramide (Glu-Cer C18:0) were increased in diabetic mouse plasma, whereas very-long-chain (C24:0, C24:1) ceramides and glucosylceramide (Glu-Cer C16:0) were decreased in diabetic mouse kidney tissue. However, circulating metabolites from the KORA study show exactly the opposite role of ceramide through SMase and genetics variants.

T2D usually occurs at the later stage of obesity, and we confirmed that lipids like SM C16:1, SM C18:1 and PC aa C38:3 could mediate the effect of BMI on T2D and also be a causal factor for T2D development. Therefore, we incorporated human genetics with mice model experiments to figure out the biological pathway. It was reported that FADS1-3 and CERS4 genetic variants with specific minor alleles (Figure 7) are associated with downregulated sphingolipids [28,33] whereas PDXDC1 and FADS2 upregulated phosphatidylcholine (Figure 7) [28], which contributes to promoting T2D pathophysiology [33,38]. CERS4 is the gene responsible for encoding ceramide synthases. Several knockout mice studies report that the inhibition of ceramide biosynthesis provokes both insulin resistance and the glucose homeostasis disruption [37,66,67]. This is contradictory with the above section which states increased ceramide causes insulin resistance. It may be attributed to that only general routes of metabolism are discussed, and specific sphingolipid species and sphingolipid metabolic pathways stay unintelligible. The function of the PDXDC1 protein, a vitamin B6-dependent decarboxylase, is not well known. It was observed in previous GWAS that PDXDC1 is linked with omega-3 (n-3) and omega-6 (n-6) polyunsaturated fatty acids (PUFAs) [68,69]. Insulin-resistance in mice induced by high-fat diets showed downregulation PDXDC1 in the liver [70]. These events suggest PDXDC1 plays a role in the fatty acids metabolism to influence phosphatidylcholine biosynthesis, regulating the risk of insulin resistance and T2D. The FADS1-3 genetic locus, which encodes FA desaturase enzymes, derive PUFAs via endogenous desaturation and elongation of fatty acids [71,72]. FADS1-3 are reported to share genome-wide significant associations with almost all cardiometabolic phenotypes such as dyslipidemia, fatty liver, obesity, and T2D [73,74,75]. The possible interpretation could be similar with PDXDC1—that the FADS genetic variants, which influence FA desaturase enzyme activity to affect sphingolipid and phosphatidylcholines biosynthesis, modulate the risking of developing T2D [76,77]. It has been observed that the FADS genes are associated with the differences in adipose tissue, body weight, and glucose homeostasis and these are regulated by PUFAs [78], which is consistent with our results that FADS1-3 have strong correlations with obesity and T2D traits in adipose, liver, and muscle tissues in ApoE−/− C57BL/6J and C3H/HeJ mice (Supplementary Figure S3). These data suggest genetic predisposition and early alterations in sphingolipids and phosphatidylcholines metabolism contribute to prediction of T2D incident.

This study has several advantages and limitations. A high number of participants were included in the study to investigate the metabolite signatures associated with obesity and T2D. We employed mediation testing to discover the novel metabolites which mediated the effect from BMI on T2D. MR tests and mice model experiments from the literature were used to establish plausible biological pathways. The most important point from this study is that lipids SM C16:1, SM C18:1, and PC aa C38:3 could be biomarkers for early stage T2D diagnosis. However, there are still some limitations that could be investigated in further studies. It is reported that storage of plasma samples for up to five years results in altered concentrations of metabolites [22] and this may influence the associations. Sphingomyelins SM C16:1 and SM C18:1 were found to be positively associated with obesity but negatively with T2D (basic model) and this is also replicated in the literature [30,31]. This could be caused from SMase converting sphingomyelins to ceramide at the later stage of obesity [63,64] and could be the reason why sphingomyelins have a positive effect on incident T2D from MR results but were negatively associated with prevalent T2D in a cross-sectional study; however, the molecular mechanism was not confirmed. Longitudinal analyses could be performed to study how metabolite concentrations change at different stages and if they are able to predict the onset of obesity related T2D. In our study, we observed sphingolipids’ metabolic pathway linked obesity and T2D but how specific metabolites SM C16:1, SM C18:1, and PC aa C38:3 work is still ambiguous and requires additional experiments to confirm more detailed molecular behavior. In the current study, metabolites were associated with BMI and T2D considering traditional covariates. Moreover, other complication factors like depressive symptoms or kidney disease or dietary intake might also have an influence on metabolic traits, which are not considered in this study.

## 5. Conclusions

This study assessed metabolic profiles from a targeted approach based on the KORA FF4 cohort. The cross-sectional analysis showed metabolic biomarkers related to obesity and T2D.For the first time, we show metabolites like SM C16:1, SM C18:1, and PC aa C38:3 performed significant mediation effects of BMI on T2D. MR analysis and mice model experiments provided new evidence in sphingolipid-driven alterations in insulin secretion and T2D development. This translates previous findings from mice models to the human metabolism. This study contributes to human validation of SM C16:1, SM C18:1, and PC aa C38:3 as biomarkers for obesity-related T2D pathophysiology that could be regarded as potential clinical targets for risk evaluation and disease monitoring. In conclusion, the findings reported here shed new light on new potential therapeutic strategies from the perspective of metabolic signatures.

## Figures and Tables

**Table 1:** Characteristics of the KORA FF4 participants based on their BMI. Mean and standard deviation are provided for quantitative variables. Count and percentage are provided for categorical variables. The significant difference of population characteristics between the individuals with obesity and the normal participants was calculated. Categorical variables were calculated via the chi square test. Student’s *t* test was used for continuous variables. Abbreviations: HbA1C, glycated hemoglobin; HDL, high-density lipoprotein; LDL, low-density lipoprotein.

BMI	Overall	Non-Obese (BMI < 30 kg/m^2^)	Obese (BMI ≥ 30 kg/m^2^)	*p* Value
Sample size	1715	1276	439	
Age mean (SD)	59.0 (12.2)	58.1 (12.1)	62.0 (12.0)	<0.001
Sex woman (%)	904 (52.7)	683 (53.5)	221 (50.3)	0.268
Weight (kg) mean (SD)	78.6 (16.0)	72.7 (11.8)	95.7 (14.2)	<0.001
Height (cm) mean (SD)	169.1 (9.6)	169.5 (9.6)	167.7 (9.7)	<0.001
Alcohol (g/day) mean (SD)	14.2 (19.4)	14.5 (18.2)	13.5 (22.5)	0.392
Waist (cm) mean (SD)	95.6 (14.0)	90.2 (10.5)	111.6 (10.3)	<0.001
Waist-hip-ratio mean (SD)	0.9 (0.1)	0.88 (0.1)	0.96 (0.1)	<0.001
Fasting glucose (mmol/L) mean (SD)	5.6 (1.3)	5.4 (1.0)	6.3 (1.7)	<0.001
2 h post glucose (mmol/L) mean (SD)	5.8 (2.2)	5.5 (1.7)	6.9 (3.2)	<0.001
Systolic blood pressure (mmHg) mean (SD)	117.9 (17.2)	116.4 (16.6)	122.5 (18.1)	<0.001
Diastolic blood pressure (mmHg) mean (SD)	72.7 (9.5)	72.2 (9.1)	74.0 (10.3)	0.001
Smoking (%)				<0.001
Smoker	267 (15.6)	221 (17.3)	46 (10.5)	
Ex-smoker	658 (38.4)	461 (36.1)	197 (44.9)	
Never-smoker	790 (46.1)	594 (46.6)	196 (44.6)	
Physical activities inactive (%)	702 (40.9)	456 (35.7)	246 (56.0)	<0.001
HDL cholesterol (mmol/L) mean (SD)	1.7 (0.5)	1.8 (0.5)	1.5 (0.4)	<0.001
LDL cholesterol (mmol/L) mean (SD)	3.5 (0.9)	3.4 (0.9)	3.6 (0.9)	0.048
Triglycerides (mmol/L) mean (SD)	1.4 (0.8)	1.25 (0.8)	1.6 (0.9)	<0.001
HbA1c (%) mean (SD)	5.5 (0.7)	5.4 (0.6)	5.8 (0.9)	<0.001
Total cholesterol (mmol/L) mean (SD)	5.6 (1.00)	5.6 (1.0)	5.5 (1.0)	0.409
C-reactive protein (mg/L) mean (SD)	2.3 (4.4)	1.7 (3.8)	3.9 (5.5)	<0.001
Type 2 diabetesy (%)	300 (17.5)	136 (10.7)	164 (37.4)	<0.001

**Table 2:** Characteristics of the KORA FF4 participants based on their diabetic status. Mean and standard deviation is provided for quantitative variables. Count and percentage are provided for categorical variables. The significant difference of population characteristics between the diabetic patients and nondiabetic participants was tested, respectively. Categorical variables were calculated via chi square test. Student’s *t* test was used for continuous variables.

Diabetes	Overall	T2D (No)	T2D (Yes)	*p* Value
Sample size	1715	1415	300	
Age mean (SD)	59.0 (12.2)	59.7 (12.2)	69.5 (10.0)	<0.001
Sex woman (%)	904 (52.7)	784 (55.4)	120 (40.0)	<0.001
Weight (kg) mean (SD)	78.6 (16.0)	76.8 (15.3)	87.2 (16.5)	<0.001
Height (cm) mean (SD)	169.1 (9.6)	169.4 (9.7)	167.2 (9.1)	<0.001
Alcohol (g/day) mean (SD)	14.2 (19.4)	13.9 (18.1)	15.8 (24.7)	0.115
Waist (cm) mean (SD)	95.6 (14.0)	93.1 (12.9)	107.8 (12.7)	<0.001
Waist-hip-ratio mean (SD)	0.9 (0.1)	0.9 (0.1)	1.0 (0.1)	<0.001
Fasting glucose (mmol/L) mean (SD)	5.6 (1.3)	5.2 (0.4)	7.6 (2.0)	<0.001
2 h post glucose (mmol/L) mean (SD)	5.8 (2.2)	5.4 (1.1)	12.6 (3.5)	<0.001
Systolic blood pressure (mmHg) mean (SD)	117.9 (17.2)	116.1 (16.2)	126.7 (18.8)	<0.001
Diastolic blood pressure (mmHg) mean (SD)	72.7 (9.5)	72.8 (9.1)	72.0 (11.1)	0.201
Smoking (%)				<0.001
Smoker	267 (15.6)	243 (17.2)	24 (8.0)	
Ex-smoker	658 (38.4)	524 (37.0)	134 (44.7)	
Never-smoker	790 (46.1)	648 (45.8)	142 (47.3)	
Physical activities inactive (%)	702 (40.9)	512 (36.2)	190 (63.3)	
HDL cholesterol (mmol/L) mean (SD)	1.72 (0.5)	1.76 (0.5)	1.48 (0.4)	<0.001
LDL cholesterol (mmol/L) mean (SD)	3.5 (0.9)	3.5 (0.9)	3.3 (0.9)	<0.001
Triglycerides (mmol/L) mean (SD)	1.4 (0.8)	1.3 (0.8)	1.8 (1.0)	<0.001
HbA1c (%) mean (SD)	5.5 (0.7)	5.3 (0.3)	6.5 (1.0)	<0.001
Total cholesterol (mmol/L) mean (SD)	5.6 (1.0)	5.6 (1.0)	5.3 (1.1)	<0.001
C-reactive protein (mg/L) mean (SD)	2.3 (4.4)	2.1 (4.3)	3.4 (4.6)	<0.001
BMI = Obese (%)	439 (25.6)	275 (19.4)	164 (54.7)	<0.001

**Table 3:** Metabolites significantly associated with BMI in the linear regression full model. The dependent variable was BMI, whereas the independent variables were the log transformed and standardized concentration of each metabolite, adjusted for age, sex, smoking status, physical activities, HDL-C, blood pressure, triglycerides, and fasting glucose. q-values were reported as *p* values adjusted for multiple testing by Bonferroni correction. Only metabolites with a *p*-value lower than 0.00034 (0.05/146) were included in this table.

	Positively Associated			
Category	Metabolite	Beta Estimate (95% CI)	*p*-value	q-value
PC aa	PC aa C38:3	1.301 (1.082–1.520)	2.50 × 10^−30^	3.65 × 10^−28^
PC aa	PC aa C38:4	0.728 (0.514–0.943)	3.74 × 10^−11^	5.47 × 10^−9^
PC aa	PC aa C40:4	0.692 (0.471–0.913)	9.89 × 10^−11^	1.44 × 10^−7^
PC aa	PC aa C32:1	0.606 (0.375–0.837)	2.93 × 10^−7^	4.28 × 10^−5^
PC aa	PC aa C40:5	0.505 (0.279–0.730)	1.19 × 10^−5^	1.74 × 10^−3^
PC aa	PC aa C36:3	0.512 (0.281–0.742)	1.41 × 10^−5^	2.06 × 10^−3^
PC aa	PC aa C36:4	0.426 (0.207–0.644)	1.38 × 10^−4^	2.01 × 10^−2^
Amino Acids	Glutamate (Glu)	1.255 (1.032–1.478)	2.09 × 10^−27^	3.05 × 10^−25^
Amino Acids	Tyrosine (Tyr)	0.901 (0.695–1.106)	1.72 × 10^−17^	2.51 × 10^−15^
Amino Acids	Phenylalanine (Phe)	0.823 (0.618–1.027)	6.11 × 10^−15^	8.92 × 10^−13^
Amino Acids	Valine (Val)	0.876 (0.652–1.100)	2.60 × 10^−14^	3.80 × 10^−12^
Amino Acids	Isoleucine (Ile)	0.866 (0.618–1.114)	1.05 × 10^−11^	1.53 × 10^−9^
Amino Acids	Leucine (Leu)	0.755 (0.515–0.995)	9.02 × 10^−10^	1.32 × 10^−7^
Amino Acids	Alanine (Ala)	0.458 (0.242–0.673)	3.27 × 10^−5^	4.78 × 10^−3^
Amino Acids	Ornithine (Orn)	0.399 (0.195–0.603)	1.30 × 10^−4^	1.90 × 10^−2^
SM	SM C16:1	1.118 (0.901–1.336)	2.65 × 10^−23^	3.87 × 10^−21^
SM	SM C18:1	1.061 (0.848–1.273)	5.81 × 10^−22^	8.48 × 10^−20^
SM	SM C20:2	0.763 (0.541–0.985)	2.14 × 10^−11^	3.12 × 10^−9^
SM	SM C18:0	0.697 (0.490–0.903)	4.52 × 10^−11^	6.60 × 10^−9^
SM	SM C24:1	0.518 (0.310–0.726)	1.16 × 10^−6^	1.69 × 10^−4^
Biogenic Amines	Alpha-Amino acid (alpha-AAA)	0.955 (0.726–1.184)	5.51 × 10^−16^	8.04 × 10^−14^
Biogenic Amines	Kynurenine	0.743 (0.524–0.962)	3.81 × 10^−11^	5.57 × 10^−9^
Biogenic Amines	4-Hydroxyproline (t4-OH-Pro)	0.485 (0.279–0.691)	4.13 × 10^−6^	6.02 × 10^−4^
Acylcarnitines	Carnitine (C0)	0.672 (0.462 -0.882)	4.20 × 10^−10^	6.13 × 10^−8^
Acylcarnitines	Valerylcarnitine (C5)	0.700 (0.478–0.922)	7.96 × 10^−10^	1.16 × 10^−7^
Acylcarnitines	Propionylcarnitine (C3)	0.670 (0.449–0.891)	3.50 × 10^−9^	5.11 × 10^−7^
Acylcarnitines	Butyrylcarnitine (C4)	0.457 (0.247–0.667)	2.15 × 10^−5^	3.14 × 10^−3^
PC ae	PC ae C36:5	0.502 (0.290–0.713)	3.49 × 10^−6^	5.09 × 10^−4^
PC ae	PC ae C36:4	0.457 (0.254–0.660)	1.07 × 10^−5^	1.56 × 10^−3^
PC ae	PC ae C32:2	0.506 (0.258–0.754)	6.52 × 10^−5^	9.52 × 10^−3^
	**Negatively Associated**			
Category	Metabolite	Beta Estimate (95% CI)	*p*-value	q-value
lysoPC	lysoPC a C17:0	−1.1 (−1.305–−0.896)	2.88 × 10^−25^	4.20 × 10^−23^
lysoPC	lysoPC a C18:2	−1.129 (−1.348–−0.911)	1.72 × 10^−23^	2.51 × 10^−21^
lysoPC	lysoPC a C18:1	−0.978 (−1.193–−0.763)	1.08 × 10^−18^	8.72 × 10^−15^
lysoPC	lysoPC a C16:0	−0.640 (−0.849–−0.432)	2.19 × 10^−9^	3.20 × 10^−7^
lysoPC	lysoPC a C18:0	−0.521 (−0.725–−0.316)	6.48 × 10^−7^	9.46 × 10^−5^
lysoPC	lysoPC a C20:4	−0.415 (−0.627–−0.203)	1.28 × 10^−4^	1.86 × 10^−2^
Amino Acids	Asparagine (Asn)	−0.642 (−0.843–−0.44)	5.30 × 10^−10^	7.73 × 10^−8^
Amino Acids	Glycine (Gly)	−0.515 (−0.724–−0.305)	1.60 × 10^−6^	2.34 × 10^−4^
PC ae	PC ae C42:3	−0.594 (−0.821–−0.368)	2.94 × 10^−7^	4.29 × 10^−5^
PC ae	PC ae C36:2	−0.607 (−0.840–−0.373)	3.75 × 10^−7^	5.48 × 10^−5^
PC ae	PC ae C40:6	−0.424 (−0.639–−0.209)	1.14 × 10^−4^	1.66 × 10^−2^
PC ae	PC ae C38:2	−0.406 (−0.613–−0.199)	1.23 × 10^−4^	1.80 × 10^−2^

**Table 4:** Metabolites significantly associated with T2D in the logistic regression full model. The dependent variable was T2D status, whereas the independent variables were the log transformed and standardized concentration of each metabolite, adjusted for age, sex, BMI, smoking status, physical activities, HDL-C, blood pressure, triglycerides, and fasting glucose. q-values were reported as *p*-values adjusted for multiple testing by Bonferroni correction. Only metabolites with *p*-value lower than 0.00034 (0.05/146) were included in this table.

Category	Metabolite	Odds Ratios (95% CI)	*p*-Value	q-Value
Acylcarnitines	Hydroxybutyrylcarnitine (C3-DC (C4-OH))	0.619 (0.363–0.888)	3.79 × 10^−6^	5.54 × 10^−4^
Biogenic Amines	Alpha-Amino acid (alpha-AAA)	0.638 (0.308–0.977)	1.77 × 10^−4^	2.58 × 10^−2^
Amino Acids	Isoleucine (Ile)	0.637 (0.293–0.987)	3.08 × 10^−4^	4.50 × 10^−2^

**Table 5:** Results for mediation analysis with the BMI as independent variable, metabolite as potential mediator, fasting glucose or HbA1c as dependent variable. q-values were reported as *p*-value adjusted for multiple testing by Bonferroni correction.

Sobel Test (Metabolite, BMI, Fasting Glucose)			Sobel Test (Metabolite, BMI, HbA1c)		
Metabolite	*p*-Value	q-Value	Metabolite	*p*-Value	q-Value
Sum of hexoses (H1)	1.49 × 10^−16^	2.18 × 10^−14^	Sum of hexoses (H1)	1.14 × 10^−15^	1.66 × 10^−13^
SM C16:1	2.88 × 10^−7^	4.20 × 10^−5^	Isoleucine (Ile)	1.08 × 10^−5^	1.58 × 10^−3^
Glutamate (Glu)	1.27 × 10^−6^	1.85 × 10^−4^	SM C16:1	1.40 × 10^−5^	2.04 × 10^−3^
PC aa C38:3	2.62 × 10^−6^	3.82 × 10^−4^	lysoPC a C18:0	5.56 × 10^−5^	8.11 × 10^−3^
lysoPC a C17:0	1.31 × 10^−5^	1.91 × 10^−3^	Leucine (Leu)	1.05 × 10^−4^	1.53 × 10^−2^
Alpha-Amino acid (alpha-AAA)	1.58 × 10^−5^	2.3 × 10^−3^	Glutamate (Glu)	1.06 × 10^−4^	1.55 × 10^−2^
Isoleucine (Ile)	1.95 × 10^−5^	2.84 × 10^−3^	lysoPC a C16:0	1.12 × 10^−4^	1.63 × 10^−2^
lysoPC a C18:0	5.00 × 10^−5^	7.30 × 10^−3^	Alpha-Amino acid (alpha-AAA)	1.48 × 10^−4^	2.16 × 10^−2^
Alanine (Ala)	6.94 × 10^−5^	1.01 × 10^−2^	PC aa C38:3	3.14 × 10^−4^	4.59 × 10^−2^
SM C18:1	1.33 × 10^−4^	1.94 × 10^−2^			
Leucine (Leu)	1.48 × 10^−4^	2.16 × 10^−2^			
SM C20:2	2.91 × 10^−4^	4.24 × 10^−2^			

## Data Availability

The KORA FF4 datasets are not publicly available but can be accessed upon application through the KORA-PASST (Project application self-service tool, https://www.helmholtz-munich.de/epi/research/cohorts/kora-cohort/data-use-and-access-via-korapasst/index.html, accessed on 13 May 2022).

## Supplementary Materials

The following supporting information can be downloaded at: https://www.mdpi.com/article/10.3390/metabo13020227/s1, Supplemental Document S1: Figure S1: Human tissue-specific gene expression and regulation. Human RNA-seq data from GTEx showing the transcript per million (TPM) expression values for the genes encoding the proteins; Figure S2. Mice tissue-specific gene expression and regulation; Figure S3. The F2 dataset which is a cross of the inbred ApoE−/− C57BL/6J and C3H/HeJ strains fed a high fat + cholesterol diet; Table S1: Complete information about all the 146 considered metabolites, including the category, abbreviations and full name used; Table S2: Association of BMI with metabolites in the basic model; Table S3: Association of BMI with metabolites in the full model; Table S4, Association of T2D with metabolites in the basic model; Table S5, Association of T2D with metabolites in the full model. Table S6, mediation test of metabolites from BMI to T2D; Table S7, mediation test of metabolite residues from BMI to T2D. Table S8: Association of BMI with metabolites in the basic model for female individuals; Table S9: Association of BMI with metabolites in the basic model for male individuals. Table S10: Association of BMI with metabolites in the full model for female individuals. Table S11: Association of BMI with metabolites in the full model for male individuals. Table S12: Association of T2D with metabolites in the basic model for female individuals; Table S13: Association of T2D with metabolites in the basic model for male individuals. Table S14: Association of T2D with metabolites in the full model for female individuals. Table S15: Association of T2D with metabolites in the full model for male individuals. Supplemental Document S2: Testing of Assumptions for Multiple Linear Regression and Logistic Regression Model. Figures S4–S19: The linearity graphs for representative metabolites for each category. Tables S16–S31: VIF values for multicollinearity between predictors in the linear regression model and logistic regression model [79,80,81,82,83,84].
