# Supplementary material for: Metabolic Signatures Elucidate the Effect of Body Mass Index on Type 2 Diabetes
Source: Metabolites. 2023 Feb 3;13(2):227. doi: 10.3390/metabo13020227 (PMC9965667; doi:10.3390/metabo13020227)
Supplement: Supplementary file 1 [file metabolites-13-00227-s001.zip › Supplemental Document S1_0501.pdf]

Metabolic Signatures Elucidate the Effect of Body Mass Index on Type 2 Diabetes

Qiuling Dong [1,2,3], Sidra Sidra [4], Christian Gieger [1,2,5], Rui Wang-Sattler [6], Wolfgang Rathmann [7], Cornelia Prehn [8], Jerzy Adamski [9,10,11], Wolfgang Koenig [12,13,14], Annette Peters [2,5,15], Harald Grallert [1,2,5,*,†] and Sapna Sharma [1,2,16,*,†]

[1] Research Unit of Molecular Epidemiology, Helmholtz Zentrum München, 85764 Neuherberg, Germany; qiuling.dong@helmholtz-muenchen.de (Q.D.); christian.gieger@helmholtz-muenchen.de (C.G.)
[2] Institute of Epidemiology, Helmholtz Zentrum München, 85764 Neuherberg, Germany; peters@helmholtz-muenchen.de
[3] Faculty of Medicine, Ludwig-Maximilians-University München, 81377 Munich, Germany
[4] Institute for Medical Information Processing, Biometry and Epidemiology (IBE), Ludwig-Maximilians-Universität München, 81377 Munich, Germany; sidra.sid@campus.lmu.de
[5] German Center for Diabetes Research (DZD), 85764 München-Neuherberg, Germany
[6] Institute of Translational Genomics, Helmholtz Zentrum München, 85764 Neuherberg, Germany;
rui.wang-sattler@helmholtz-muenchen.de
[7] Institute for Biometrics and Epidemiology, German Diabetes Center, Leibniz Center for Diabetes Research, Heinrich Heine University Düsseldorf, 40225 Düsseldorf, Germany; rathmann@ddz.de
[8] Metabolomics and Proteomics Core Facility, Helmholtz Zentrum München, 85764 Neuherberg, Germany; prehn@helmholtz-muenchen.de
[9] Institute of Experimental Genetics, Helmholtz Zentrum München, German Research Center for
Environmental Health, Ingolstädter Landstraße 1, 85764 Neuherberg, Germany; info.adamski@gmx.org
[10] Department of Biochemistry, Yong Loo Lin School of Medicine, National University of Singapore, 8 Medical Drive, Singapore 117597, Singapore
[11] Institute of Biochemistry, Faculty of Medicine, University of Ljubljana, Vrazov trg 2, 1000 Ljubljana, Slovenia
[12] German Research Center for Cardiovascular Disease (DZHK), Partner site Munich Heart Alliance,
81377 Munich, Germany; koenig@dhm.mhn.de
[13] Deutsches Herzzentrum München, Technische Universität München, 81377 Munich, Germany
[14] Institute of Epidemiology and Medical Biometry, University of Ulm, 89069 Ulm, Germany
[15] Chair of Epidemiology, Faculty of Medicine, Ludwig-Maximilians-University München, 81377 Munich, Germany
[16] Chair of Food Chemistry and Molecular Sensory Science, Technical University of Munich, 85354 Freising-Weihenstephan, Germany
* Correspondence: harald.grallert@helmholtz-muenchen.de (H.G.); sapna.sharma@tum.de (S.S.)
† These authors contributed equally to this work.

Supplementary Material

Supplementary table S1: Complete information about all the 146 considered metabolites
Complete information about all the 146 considered metabolites, including the category, abbreviations and full name used.

Supplementary table S2: Association of BMI with metabolites in the basic model
Description: Association statistics for the association of BMI with metabolites in a linear regression model lm(BMI ~ metabolite + age + sex).

Supplementary table S3: Association of BMI with metabolites in the full model
Description: Association statistics for the association of BMI with metabolites in a linear regression model lm(BMI ~ metabolite + age + sex + physical activity + smoking + systolic blood pressure + HDL-C + triglyceride + fasting glucose).

Supplementary table S4: Association of T2D with metabolites in the basic model
Description: Association statistics for the association of T2D with metabolites in a logistic regression model glm(T2D ~ metabolite + age + sex + BMI).

Supplementary table S5: Association of T2D with metabolites in the full model
Description: Association statistics for the association of T2D with metabolites in a logistic regression model glm(T2D ~ metabolite + age + sex + BMI + physical activity + smoking + systolic blood pressure + HDL-C + triglyceride + fasting glucose).

Supplementary table S6: Mediation test of metabolite residues from BMI to fasting glucose
Description: Metabolite residue was calculated from a linear regression model lm (metabolite ~ age + sex + physical activity + smoking + systolic blood pressure + HDL-C + triglyceride) and residue function lm$residuals. Mediation test statistics for residuals' influence of BMI on fasting glucose in a mediation.test (metabolite residue, BMI, fasting glucose).

Supplementary table S7: Mediation test of metabolite residues from BMI to HbA1c
Description: Metabolite residue was calculated from a linear regression model lm (metabolite ~ age + sex + physical activity + smoking + systolic blood pressure + HDL-C + triglyceride) and residue function lm$residuals. Mediation test statistics for residuals' influence of BMI on fasting glucose in a mediation.test (metabolite residue, BMI, HbA1c).

Sensitivity Analysis
Sensitivity analysis was conducted by gender stratification. Female and male participants were separated to estimate the associations with BMI and T2D. The full statistics were shown in **Supplementary Table S8-S15**. Metabolites like SM C16:1, SM C18:1 and PC aa C38:3 were significantly associated with BMI in both men and women individuals (basic and full models, Table S8-S11). SM C16:1 were significantly related to

T2D in female participants, SM C18.1 was slightly associated with T2D (p-value = 0.075). Both SM C16:1 and SM C18:1 were significantly related to T2D in male participants (basic model, Table S12-S13). Generally there is not much gender difference in finding metabolites.

Supplementary table S8: Association of BMI with metabolites in the basic model for female individuals

Description: Association statistics for the association of BMI with metabolites in a linear regression model lm(BMI ~ metabolite + age).

Supplementary table S9: Association of BMI with metabolites in the basic model for male individuals

Description: Association statistics for the association of BMI with metabolites in a linear regression model lm(BMI ~ metabolite + age).

Supplementary table S10: Association of BMI with metabolites in the full model for female individuals

Description: Association statistics for the association of BMI with metabolites in a linear regression model lm(BMI ~ metabolite + age + physical activity + smoking + systolic blood pressure + HDL-C + triglyceride + fasting glucose).

Supplementary table S11: Association of BMI with metabolites in the full model for male individuals

Description: Association statistics for the association of BMI with metabolites in a linear regression model lm(BMI ~ metabolite + age + physical activity + smoking + systolic blood pressure + HDL-C + triglyceride + fasting glucose).

Supplementary table S12: Association of T2D with metabolites in the basic model for female individuals

Description: Association statistics for the association of T2D with metabolites in a logistic regression model glm(T2D ~ metabolite + age + BMI).

Supplementary table S13: Association of T2D with metabolites in the basic model for male individuals

Description: Association statistics for the association of T2D with metabolites in a logistic regression model glm(T2D ~ metabolite + age + BMI).

Supplementary table S14: Association of T2D with metabolites in the full model for female individuals

Description: Association statistics for the association of T2D with metabolites in a logistic regression model glm(T2D ~ metabolite + age + BMI + physical activity + smoking + systolic blood pressure + HDL-C + triglyceride + fasting glucose).

Supplementary table S15: Association of T2D with metabolites in the full model for male individuals

Description: Association statistics for the association of T2D with metabolites in a logistic regression model glm(T2D ~ metabolite + age + BMI + physical activity + smoking + systolic blood pressure + HDL-C + triglyceride + fasting glucose).

Gene expression analysis

We employed the Genotype-Tissue Expression (GTEx) human database and the Mouse Genome Informatics (MGI) database to investigate the tissue-specific role of the identified genes CERS4, PDXDC1 and FADS1-3. Human expression data presented were generated through a multi-gene query on the GTEx portal on 17/10/2022 from https://www.gtexportal.org/home/multiGeneQueryPage. Mice expression data was screened on the GXD portal and visualized using the Morpheus heat map and analysis tool created by the Broad Institute from http://www.informatics.jax.org/expression.shtml. While the functional role of a subset of these genes may be more global, others may imply specific pathways. For instance, in human tissues, CERS4, FADS1, FADS2 and PDXDC1 were predominantly expressed in the thyroid, adrenal, brain, and testis tissues. FADS3, showed more sporadic expression in relevant human tissues, in adipose and artery tissues. In mice, FADS1 and FADS2 were primarily expressed in the liver. FADS1-3 have been identified as genetic risk factors for obesity or T2D, preferentially associated with abnormal lipid levels like fatty acids, cholesterol and triglyceride [1,2].

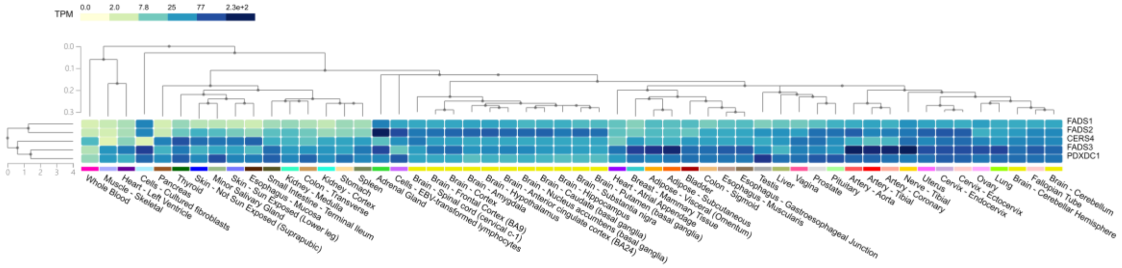

Figure S1. Human tissue-specific gene expression and regulation. Human-seq data from GTEx showing the transcript per million (TPM) expression values for the genes encoding the proteins. Data is arranged into two clusters.

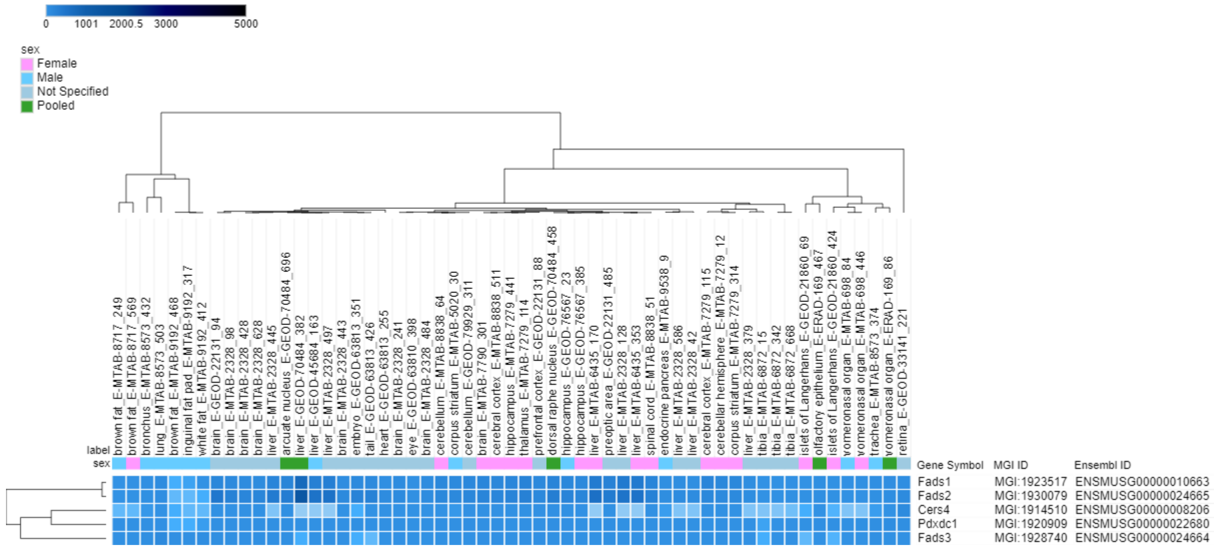

Figure S2. Mice tissue-specific gene expression and regulation. RNA-Seq tissue specific gene expression and regulation in mice for the C57BL/6J strain from the MGI database. The expression values for the genes encoding the proteins are shown in transcript per million (TPM). Data is only shown for the available genes in the respective databases.

Identifying the tissue-specific role of SM C16:1, SM C18:1 and PC aa C38:3 associated gene using mouse databases

Mouse orthologs were identified for the 5 genes (FADS1-3, CERS4 and PDXDC1) associated with SM C16:1, SM C18:1 and PC aa C38:3 using the Mouse Genome Database [3]. The reference mouse database was used to identify gene expression correlations with relevant obesity and T2D traits in mice adipose, liver, muscle and brain tissue: an F2 cross of the inbred ApoE−/− C57BL/6J and C3H/HeJ strains (n = 334 mice that were fed with a high fat diet from 8 to 16 weeks of age and euthanized at 24 weeks of age) [4]. The adipose, liver, muscle and brain tissue expression data of the 334 F2 cross mice was accessed using the publicly available dataset Sage BioNetworks at https://www.synapse.org/#!Synapse:syn4497 [4]. 3 of the genes had mouse orthologs. The weight mild correlation coefficients (bicor) measures samples based on the median and are less sensitive to outliers, which provides a robust alternative to similarity metrics. WGCNA R package was used to calculate the bicor coefficients and the p values for the association of gene expression levels and the selected relevant traits [5]. We observed correlations between adipose, liver and muscle gene expression and numerous essential obesities and T2D traits, including weight, body fat composition, LDL, HDL, cholesterol, triglycerides, insulin, glucose, etc. We noticed FADS1 strong enrichment in muscle tissue, FADS2 in liver tissue and FADS3 in adipose and liver tissues for obesity and T2D traits. In general, FADS1-3 have strong correlations with obesity and T2D traits in adipose, liver and muscle tissues, which indicated these genes may regulate the pathways of obesity to T2D development.

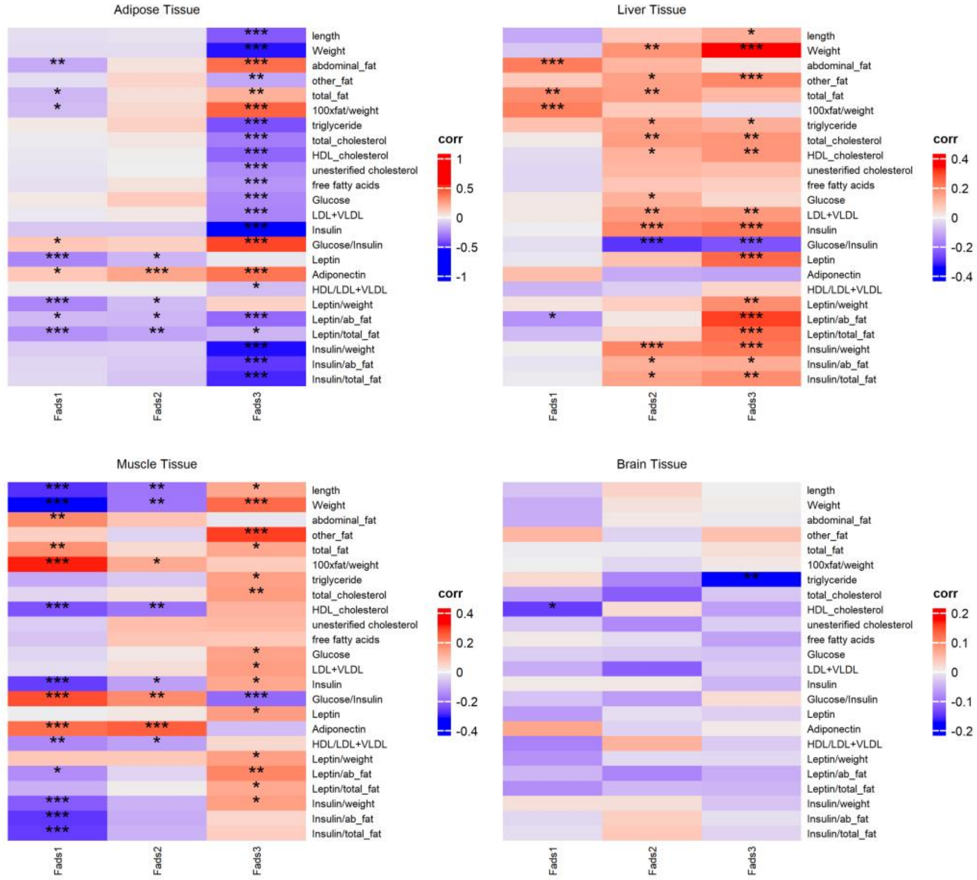

Figure S3. The F2 dataset which is a cross of the inbred ApoE−/− C57BL/6J and C3H/HeJ strains fed a high fat + cholesterol diet. The significance of the correlations R^2 is as indicated (*** for $p < 0.001$, ** for $p < 0.01$ and * for $p < 0.05$)

References

1. Stancakova, A.; Paananen, J.; Soininen, P.; Kangas, A.J.; Bonnycastle, L.L.; Morken, M.A.; Collins, F.S.; Jackson, A.U.; Boehnke, M.L.; Kuusisto, J.; et al. Effects of 34 risk loci for type 2 diabetes or hyperglycemia on lipoprotein subclasses and their composition in 6,580 nondiabetic Finnish men. *Diabetes* **2011**, *60*, 1608-1616, doi:10.2337/db10-1655.
2. Khamlaoui, W.; Mehri, S.; Hammami, S.; Hammouda, S.; Chraeif, I.; Elosua, R.; Hammami, M. Association Between Genetic Variants in FADS1-FADS2 and ELOVL2 and Obesity, Lipid Traits, and Fatty Acids in Tunisian Population. *Clin Appl Thromb-Hem* **2020**, *26*, doi:Artn 1076029620915286

10.1177/1076029620915286.

3. Bult, C.J.; Blake, J.A.; Smith, C.L.; Kadin, J.A.; Richardson, J.E.; Anagnostopoulos, A.; Asabor, R.; Baldarelli, R.M.; Beal, J.S.; Bello, S.M.; et al. Mouse Genome Database (MGD) 2019. *Nucleic Acids Res* **2019**, *47*, D801-D806, doi:10.1093/nar/gky1056.
4. Yang, X.; Schadt, E.E.; Wang, S.; Wang, H.; Arnold, A.P.; Ingram-Drake, L.; Drake, T.A.; Lusis, A.J. Tissue-specific expression and regulation of sexually dimorphic genes in mice. *Genome Res* **2006**, *16*, 995-1004, doi:10.1101/gr.5217506.

5. Langfelder, P.; Horvath, S. WGCNA: an R package for weighted correlation network analysis. *Bmc Bioinformatics* **2008**, *9*, doi:Artn 559

10.1186/1471-2105-9-559.
