# Supplementary material for: Metabolic Signatures Elucidate the Effect of Body Mass Index on Type 2 Diabetes
Source: Metabolites. 2023 Feb 3;13(2):227. doi: 10.3390/metabo13020227 (PMC9965667; doi:10.3390/metabo13020227)
Supplement: Supplementary file 1 [file metabolites-13-00227-s001.zip › Supplemental Document S2_0501.pdf]

Metabolic Signatures Elucidate the Effect of Body Mass Index on Type 2 Diabetes

Qiuling Dong [1,2,3], Sidra Sidra [4], Christian Gieger [1,2,5], Rui Wang-Sattler [6], Wolfgang Rathmann [7], Cornelia Prehn [8], Jerzy Adamski [9,10,11], Wolfgang Koenig [12,13,14], Annette Peters [2,5,15], Harald Grallert [1,2,5,*,†] and Sapna Sharma [1,2,16,*,†]

[1] Research Unit of Molecular Epidemiology, Helmholtz Zentrum München, 85764 Neuherberg, Germany; qiuling.dong@helmholtz-muenchen.de (Q.D.); christian.gieger@helmholtz-muenchen.de (C.G.)

[2] Institute of Epidemiology, Helmholtz Zentrum München, 85764 Neuherberg, Germany; peters@helmholtz-muenchen.de

[3] Faculty of Medicine, Ludwig-Maximilians-University München, 81377 Munich, Germany

[4] Institute for Medical Information Processing, Biometry and Epidemiology (IBE), Ludwig-Maximilians-Universität München, 81377 Munich, Germany; sidra.sid@campus.lmu.de

[5] German Center for Diabetes Research (DZD), 85764 München-Neuherberg, Germany

[6] Institute of Translational Genomics, Helmholtz Zentrum München, 85764 Neuherberg, Germany; rui.wang-sattler@helmholtz-muenchen.de

[7] Institute for Biometrics and Epidemiology, German Diabetes Center, Leibniz Center for Diabetes Research, Heinrich Heine University Düsseldorf, 40225 Düsseldorf, Germany; rathmann@ddz.de

[8] Metabolomics and Proteomics Core Facility, Helmholtz Zentrum München, 85764 Neuherberg, Germany; prehn@helmholtz-muenchen.de

[9] Institute of Experimental Genetics, Helmholtz Zentrum München, German Research Center for Environmental Health, Ingolstädter Landstraße 1, 85764 Neuherberg, Germany; info.adamski@gmx.org

[10] Department of Biochemistry, Yong Loo Lin School of Medicine, National University of Singapore, 8 Medical Drive, Singapore 117597, Singapore

[11] Institute of Biochemistry, Faculty of Medicine, University of Ljubljana, Vrazov trg 2, 1000 Ljubljana, Slovenia

[12] German Research Center for Cardiovascular Disease (DZHK), Partner site Munich Heart Alliance, 81377 Munich, Germany; koenig@dhm.mhn.de

[13] Deutsches Herzzentrum München, Technische Universität München, 81377 Munich, Germany

[14] Institute of Epidemiology and Medical Biometry, University of Ulm, 89069 Ulm, Germany

[15] Chair of Epidemiology, Faculty of Medicine, Ludwig-Maximilians-University München, 81377 Munich, Germany

[16] Chair of Food Chemistry and Molecular Sensory Science, Technical University of Munich, 85354 Freising-Weihenstephan, Germany

* Correspondence: harald.grallert@helmholtz-muenchen.de (H.G.); sapna.sharma@tum.de (S.S.)

† These authors contributed equally to this work.

Supplementary Material

Testing of Assumptions for Multiple Linear Regression and Logistic Regression Model:

lm(BMI ~ metabolite + age + sex + physical activity + smoking + systolic blood pressure + HDL-C + triglyceride + fasting glucose)

glm(T2D ~ metabolite + age + sex + BMI + physical activity + smoking + systolic blood pressure + HDL-C + triglyceride + fasting glucose)

Basic assumptions of multiple linear regression and logistic regression models were tested for one member of each metabolite specie assuming that a member of one metabolite class sufficiently represented the entire class.

For each metabolite, a separate multiple linear regression model was fitted taking continuous BMI as dependent variable and each of the 146 metabolites as independent variable adjusted for the selected covariates. The basic assumptions of multiple linear regression were tested and satisfied. The diagnostic plots for linear regression were visually inspected. Assumptions of normal distribution of residuals and heteroskedasticity were deemed to be approximately satisfied. **The linearity graphs for representative metabolites are presented below as Figure S4-Figure S11**.

For the logistic regression model, binary diabetes status (yes/no) is the dependent variable and each of the 146 metabolites as an independent variable adjusted for the selected covariates. The basic assumptions of logistic regression were tested and satisfied. The assumption of linearity between continuous predictor variables and the logit of the outcome was tested by analyzing linearity graphs (**Figure S12-Figure S19**). All metabolites seemed to have an approximately linear relationship with the logit of the outcome i.e. Diabetes Status. The assumption of absence of influential points was assumed to have been fulfilled as the data were pre-processed and the natural log-transformed.

For both models, the assumption of absence of multicollinearity was tested using function "vif" of R package "car" employing again one member per class rule. This function gives values of Variance Inflation Factor (VIF) which is a measure of the magnitude of multicollinearity in regression analysis. Multicollinearity exists when there is a correlation between multiple independent variables in a multiple regression model. A value of 1 indicates no collinearity between an independent variable and other predictors of the model whereas infinity suggests perfect collinearity. As a general rule, a VIF value greater than 5 or 10 indicates a serious collinearity problem [1]. This assumption was also deemed to have been satisfied sufficiently as Variance Inflation Factor (VIF) values for nearly all of the predictor variables were close to 1 implying that there is no to very little multicollinearity. **VIF values for multicollinearity between predictors in both models are presented below (Table S16-Table S31).**

Abbreviations: BMI: Body Mass Index; HDL-C: High Density Lipoprotein Cholesterol;

Diagnostic Plots and Tables for Multiple Linear Regression Model:

Figure S4: The linearity graphs for representative metabolites carnitine (C0) of acylcarnitines:

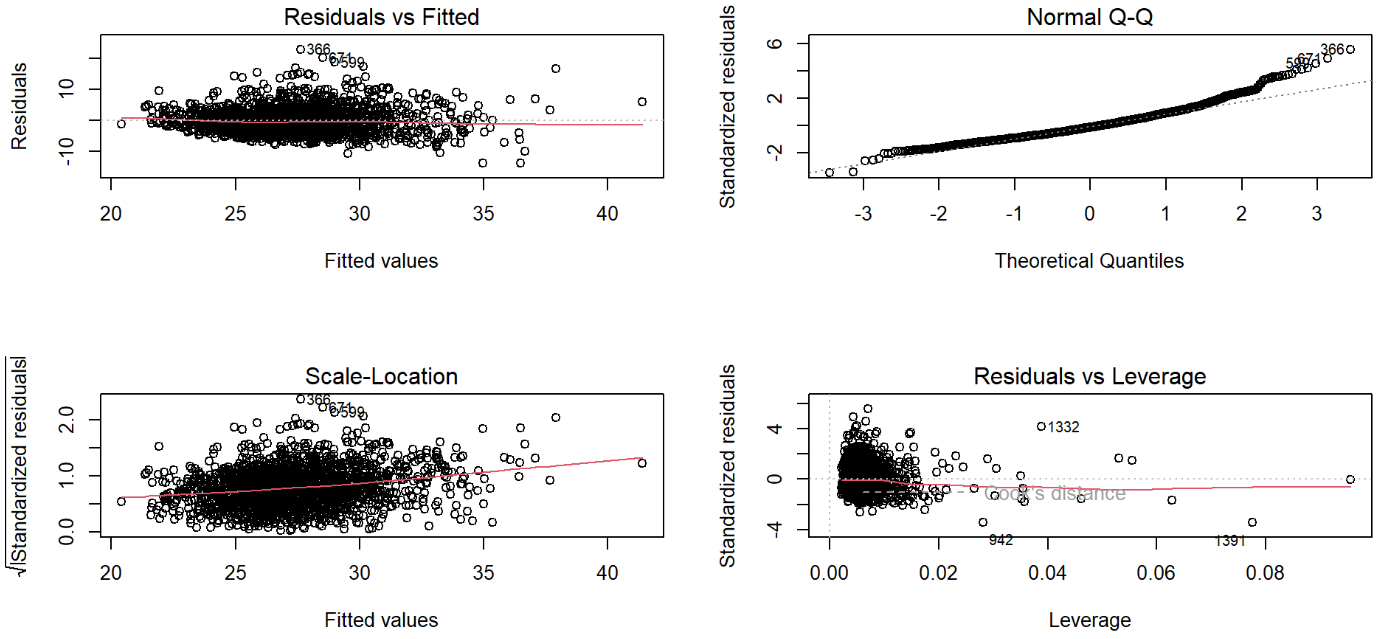

Table S16: VIF values for multicollinearity between predictors in the model for acylcarnitines-carnitine (C0)

Predictors	GVIF	Df	GVIF^(1/(2*Df))
Carnitine	1.16	1	1.08
Age	1.23	1	1.11
Sex	1.39	1	1.18
Smoking	1.10	2	1.03
Physical activity	1.06	1	1.03
HDL-C	1.59	1	1.26
Systolic Blood Pressure	1.24	1	1.11
Triglycerides	1.40	1	1.18
Fasting serum glucose	1.26	1	1.12

Figure S5: The linearity graphs for representative metabolites lysoPC a C16:0 of lysophosphatidylcholines

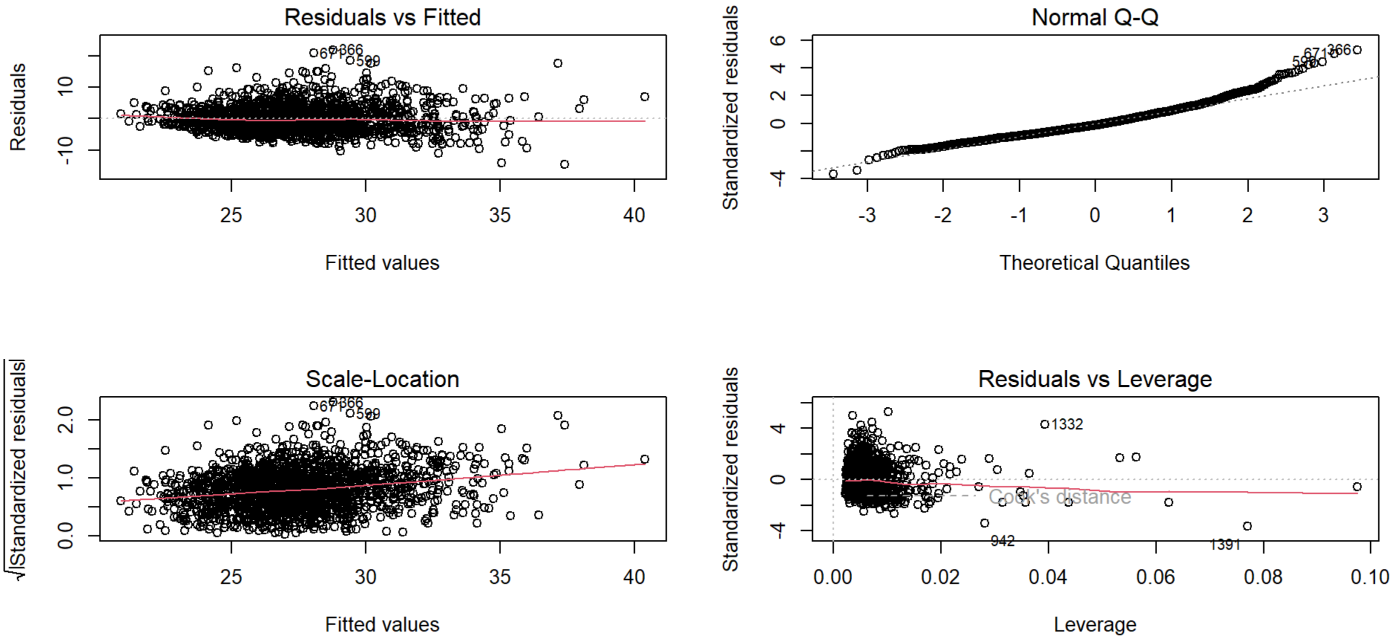

Table S17: VIF values for multicollinearity between predictors in the model for lysophosphatidylcholines - lysoPC a C16:0

Predictors	GVIF	Df	GVIF^(1/(2*Df))
lysoPC a C16:0	1.12	1	1.06
Age	1.18	1	1.08
Sex	1.42	1	1.19
Smoking	1.09	2	1.02
Physical activity	1.06	1	1.03
HDL-C	1.61	1	1.27
Systolic Blood Pressure	1.24	1	1.11
Triglycerides	1.45	1	1.20
Fasting serum glucose	1.29	1	1.14

Figure S6: The linearity graphs for representative metabolites PC aa C28:1 of diacylphosphatidylcholines

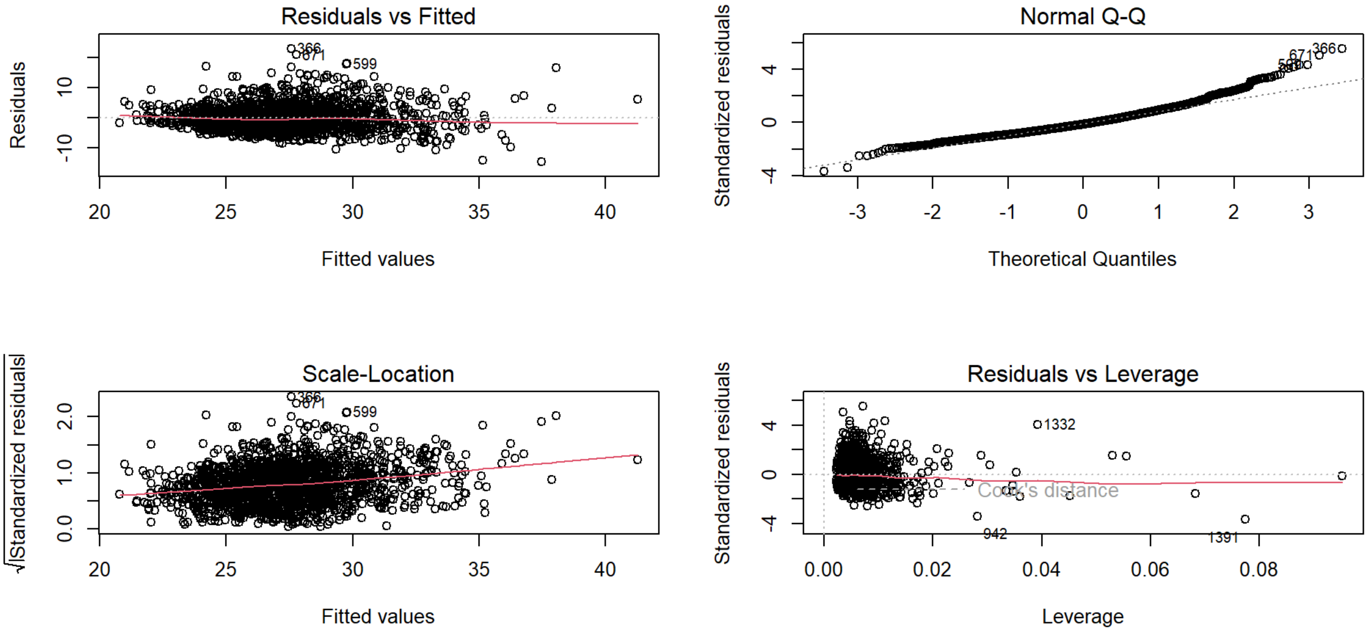

Table S18: VIF values for multicollinearity between predictors in the model for diacylphosphatidylcholines - PC aa C28:1

Predictors	GVIF	Df	GVIF^(1/(2*Df))
PC aa C28:1	1.26	1	1.12
Age	1.18	1	1.09
Sex	1.38	1	1.18
Smoking	1.11	2	1.03
Physical activity	1.06	1	1.03
HDL-C	1.72	1	1.31
Systolic Blood Pressure	1.24	1	1.11
Triglycerides	1.44	1	1.20
Fasting serum glucose	1.27	1	1.13

Figure S7: The linearity graphs for representative metabolites PC ae C30:0 of acylalkylphosphatidylcholine

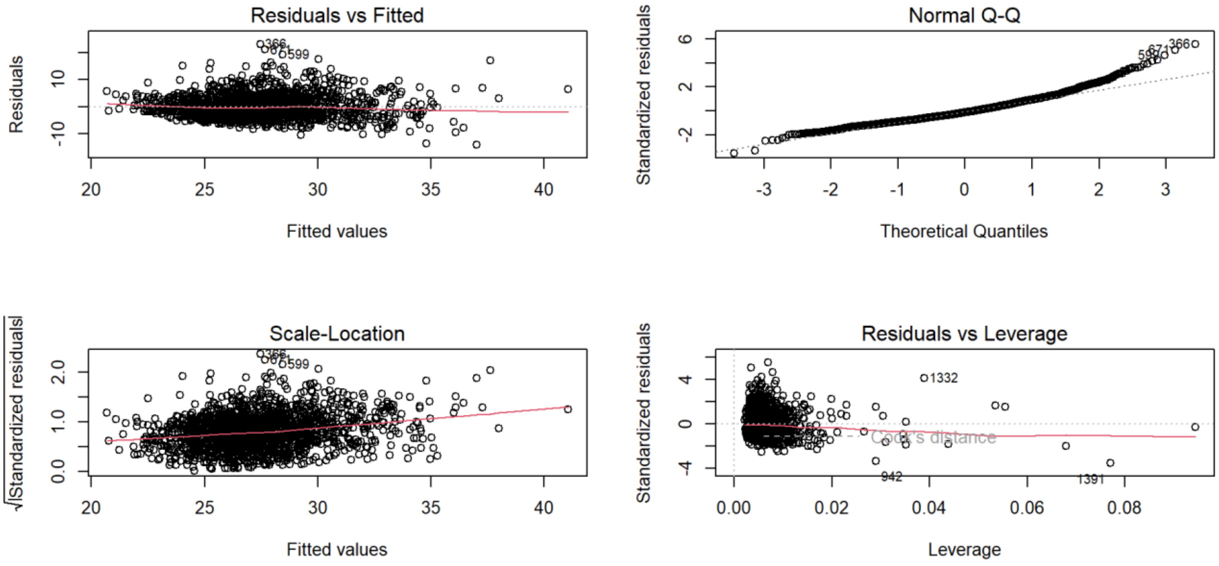

Table S19: VIF values for multicollinearity between predictors in the model for acylalkylphosphatidylcholine - PC ae C30:0

Predictors	GVIF	Df	GVIF^(1/(2*Df))
PC ae C30:0	1.24	1	1.11
Age	1.17	1	1.08
Sex	1.37	1	1.17
Smoking	1.11	2	1.03
Physical activity	1.06	1	1.03
HDL-C	1.68	1	1.30
Systolic Blood Pressure	1.24	1	1.11
Triglycerides	1.40	1	1.18
Fasting serum glucose	1.28	1	1.13

Figure S8: The linearity graphs for representative metabolite SM (OH) C14:1 of sphingomyelins

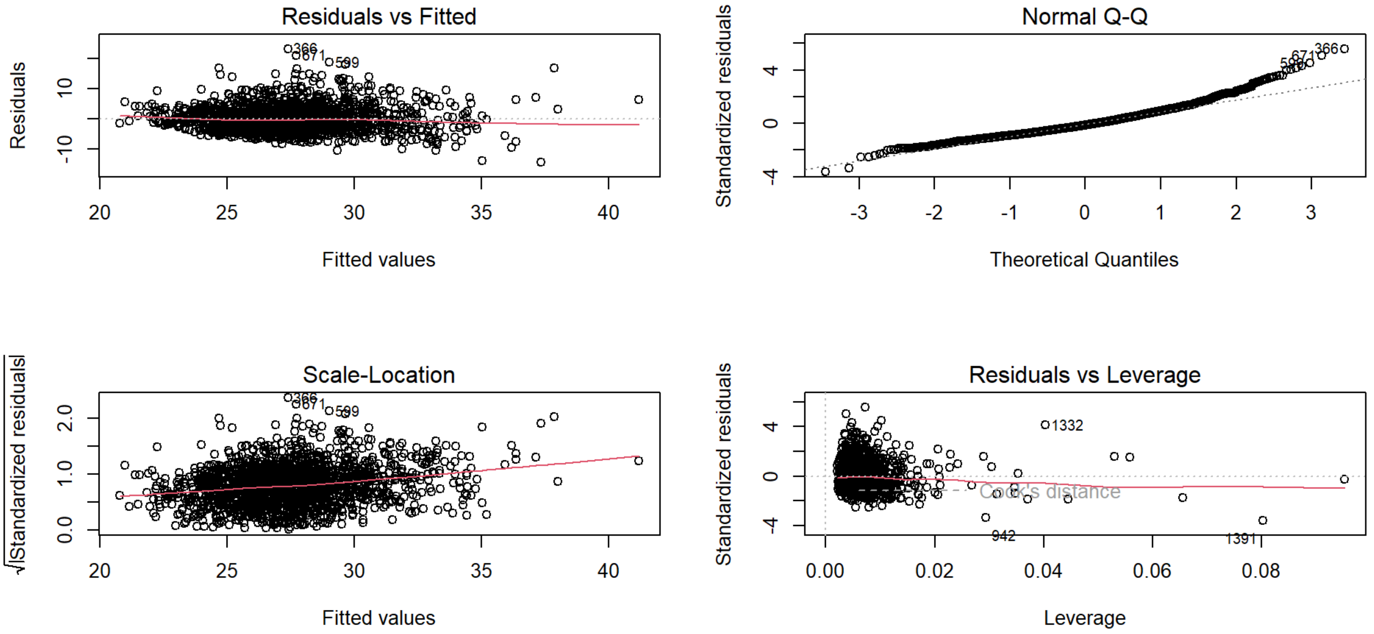

Table S20: VIF values for multicollinearity between predictors in the model for sphingomyelins - SM (OH) C14:1

Predictors	GVIF	Df	GVIF^(1/(2*Df))
SM (OH) C14:1	1.31	1	1.15
Age	1.20	1	1.09
Sex	1.41	1	1.19
Smoking	1.12	2	1.03
Physical activity	1.06	1	1.03
HDL-C	1.62	1	1.27
Systolic Blood Pressure	1.24	1	1.11
Triglycerides	1.39	1	1.18
Fasting serum glucose	1.28	1	1.13

Figure S9: The linearity graphs for representative metabolite Hexoses (H1)

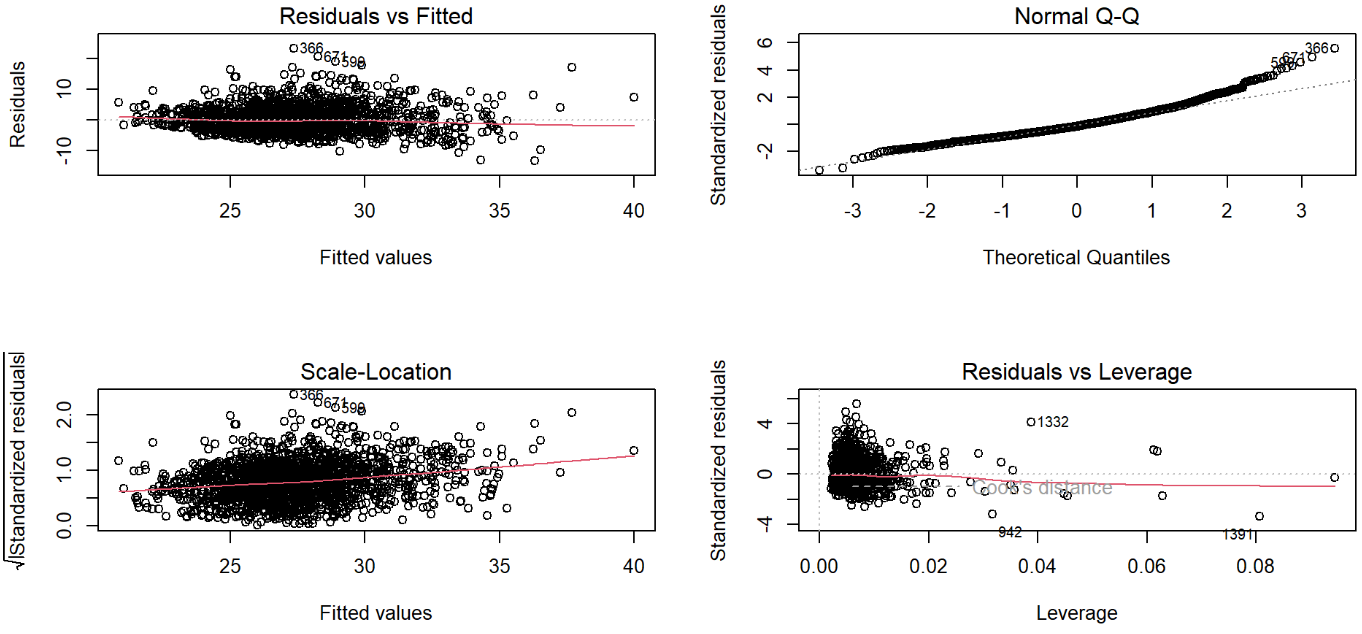

Table S21: VIF values for multicollinearity between predictors in the model for Hexoses (H1)

Predictors	GVIF	Df	GVIF^(1/(2*Df))
Hexoses	3.92	1	1.98
Age	1.18	1	1.09
Sex	1.35	1	1.16
Smoking	1.10	2	1.02
Physical activity	1.06	1	1.03
HDL-C	1.60	1	1.26
Systolic Blood Pressure	1.24	1	1.11
Triglycerides	1.39	1	1.18
Fasting serum glucose	3.94	1	1.98

Figure S10: The linearity graphs for representative metabolite alanine (Ala) for amino acids

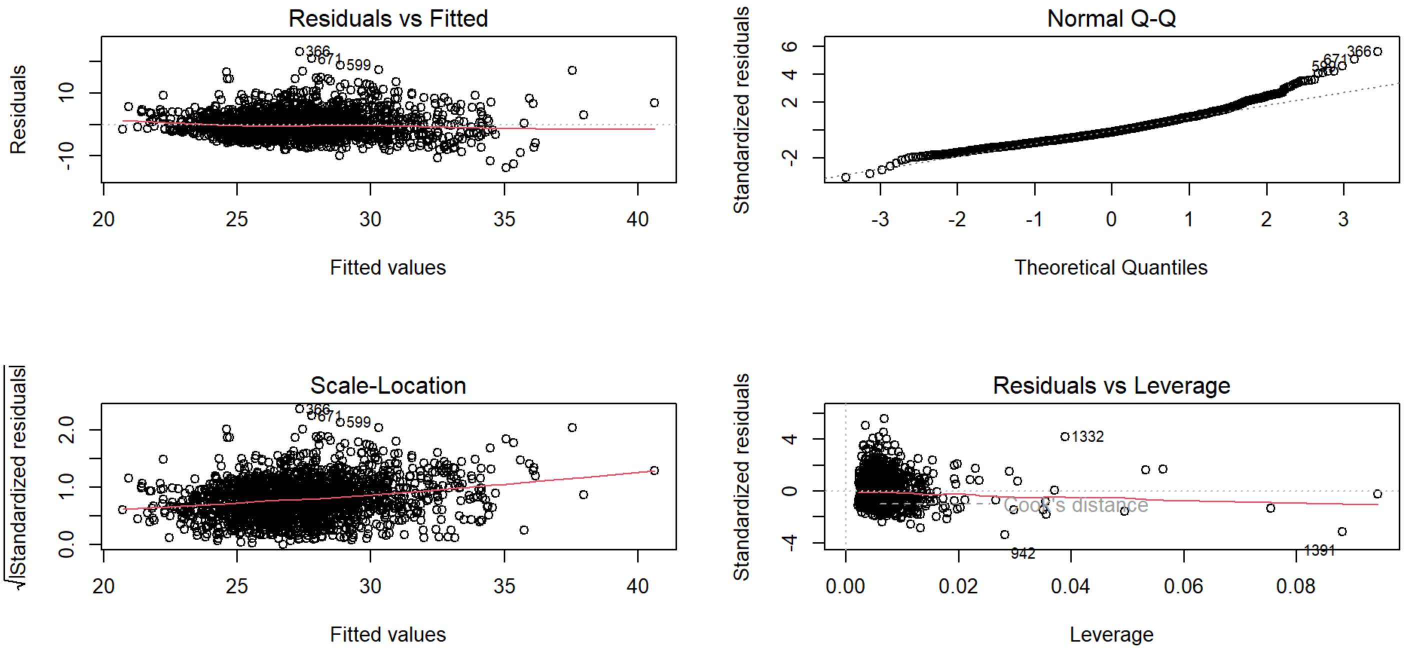

Table S22: VIF values for multicollinearity between predictors in the model for amino acids - alanine (Ala)

Predictors	GVIF	Df	GVIF^(1/(2*Df))
Alanine	1.19	1	1.09
Age	1.19	1	1.09
Sex	1.34	1	1.16
Smoking	1.10	2	1.02
Physical activity	1.06	1	1.03
HDL-C	1.59	1	1.26
Systolic Blood Pressure	1.24	1	1.11
Triglycerides	1.41	1	1.19
Fasting serum glucose	1.32	1	1.15

Figure S11: The linearity graphs for representative metabolite acetylornithine (Ac-Orn) for biogenic amines

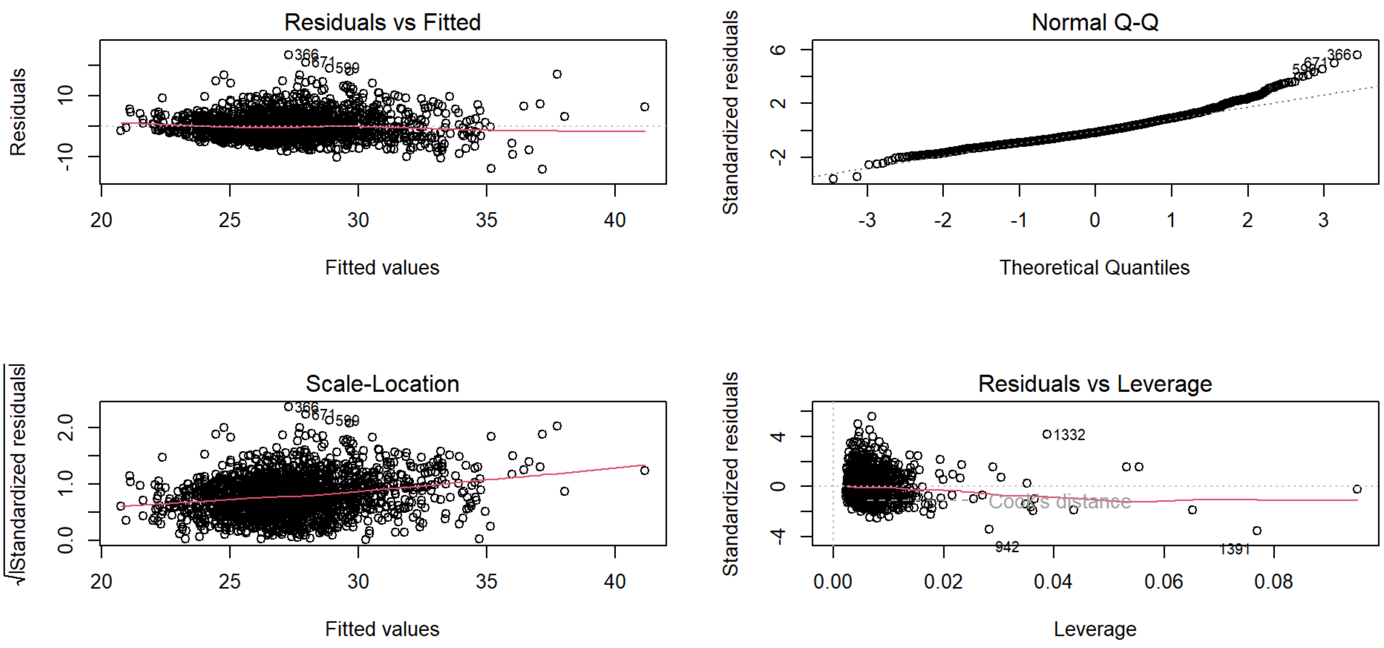

Table S23: VIF values for multicollinearity between predictors in the model for biogenic amines - acetylornithine

Predictors	GVIF	Df	GVIF^(1/(2*Df))
Acetylornithine	1.06	1	1.03
Age	1.23	1	1.11
Sex	1.35	1	1.16
Smoking	1.10	2	1.02
Physical activity	1.06	1	1.03
HDL-C	1.58	1	1.26
Systolic Blood Pressure	1.24	1	1.11
Triglycerides	1.39	1	1.18
Fasting serum glucose	1.26	1	1.12

Diagnostic Plots and Tables for Multiple Linear Regression Model:

Figure S12: The linearity graphs for representative metabolite carnitine (C0) for acylcarnitines

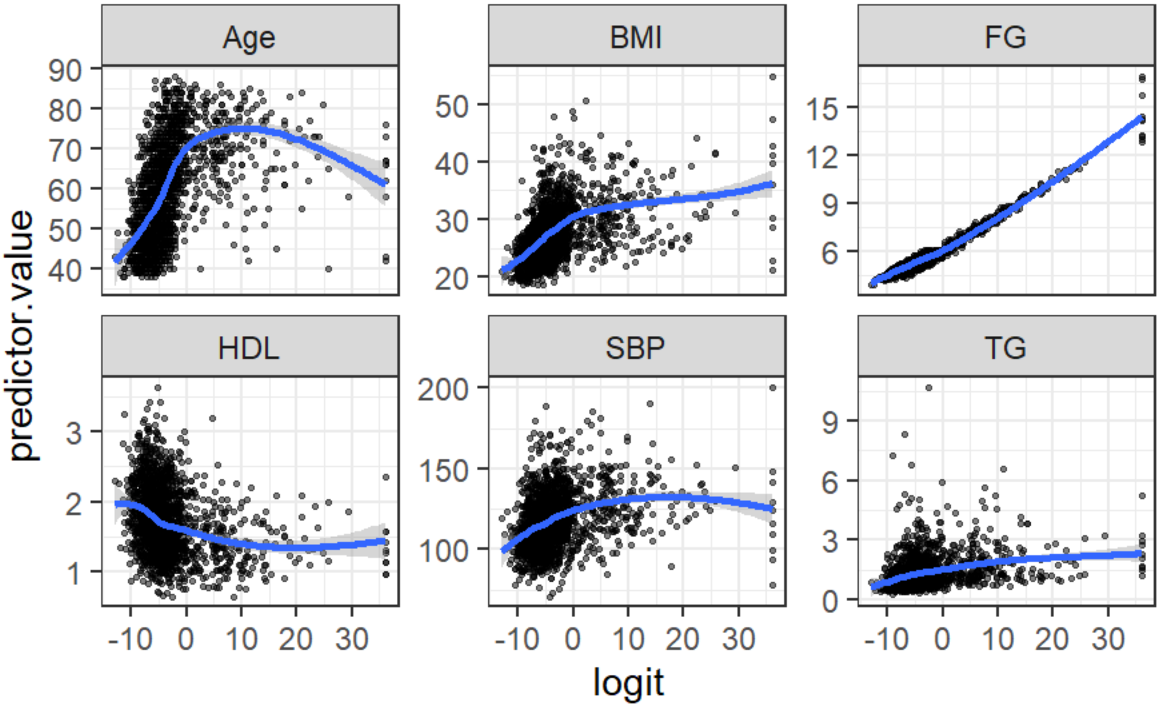

Table S24: VIF values for multicollinearity between predictors in the model for acylcarnitines - carnitine (C0)

Predictors	GVIF	Df	GVIF^(1/(2*Df))
Carnitine	1.13	1	1.06
Age	1.15	1	1.07
Sex	1.52	1	1.23
BMI	1.19	1	1.09
Smoking	1.37	2	1.08
Physical activity	1.07	1	1.04
HDL-C	1.70	1	1.30
Systolic Blood Pressure	1.13	1	1.06
Triglycerides	1.39	1	1.18
Fasting serum glucose	1.10	1	1.05

Figure S13: The linearity graphs for representative metabolite lysoPC a C16:0 for lysophosphatidylcholines

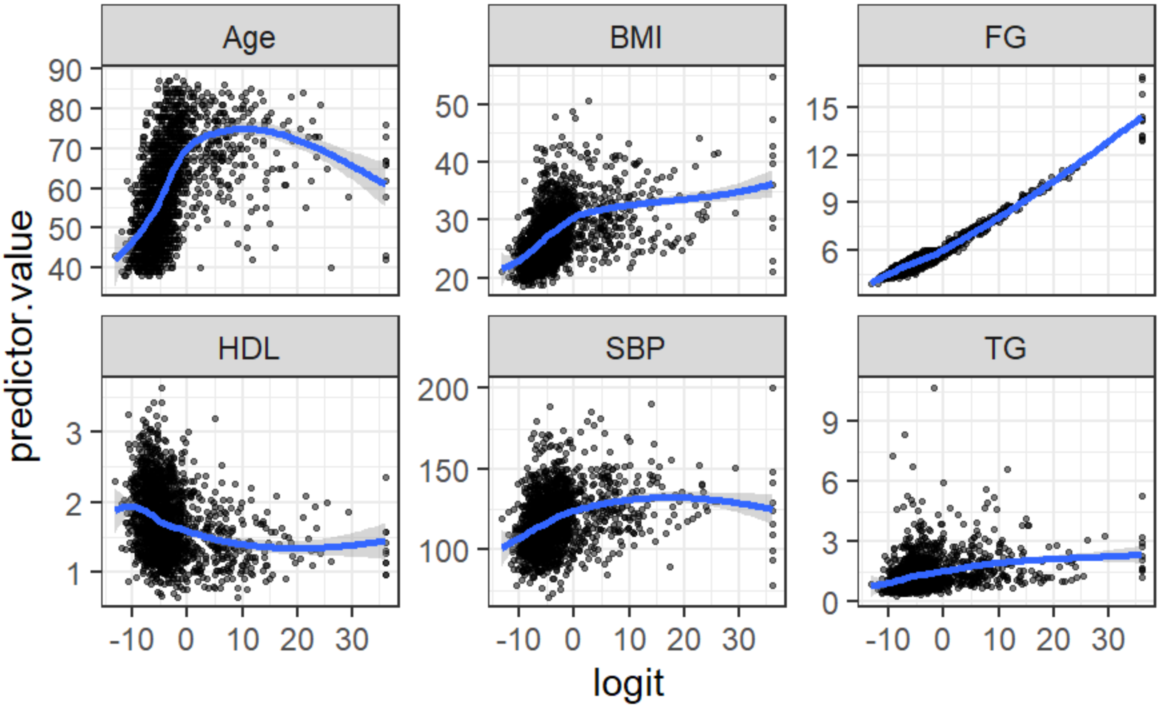

Table S25: VIF values for multicollinearity between predictors in the model for lysophosphatidylcholines - lysoPC a C16:0

Predictors	GVIF	Df	GVIF^(1/(2*Df))
lysoPC a C16:0	1.15	1	1.07
Age	1.14	1	1.07
Sex	1.53	1	1.24
BMI	1.22	1	1.10
Smoking	1.38	2	1.08
Physical activity	1.07	1	1.03
HDL-C	1.71	1	1.31
Systolic Blood Pressure	1.12	1	1.06
Triglycerides	1.43	1	1.20
Fasting serum glucose	1.10	1	1.05

Figure S14: The linearity graphs for representative metabolite PC aa C28:1 for diacylphosphatidylcholines

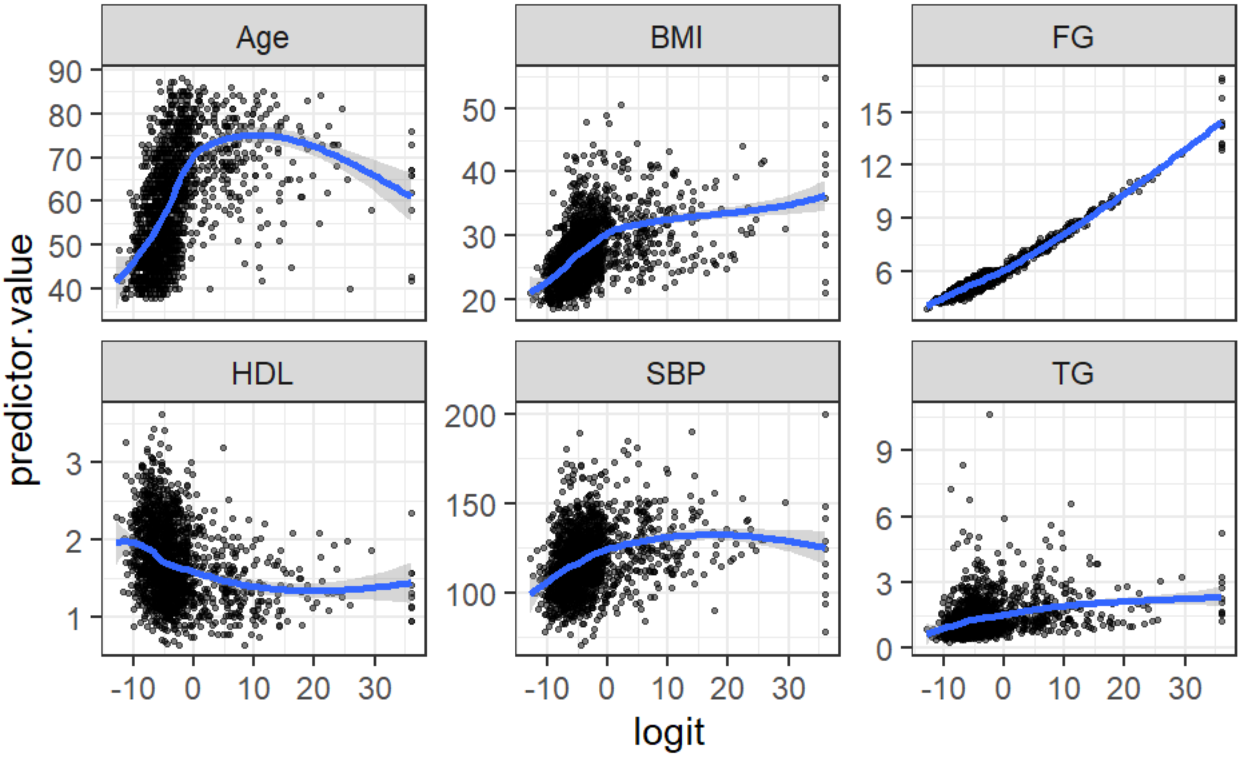

Table S26: VIF values for multicollinearity between predictors in the model for diacylphosphatidylcholines - PC aa C28:1

Predictors	GVIF	Df	GVIF^(1/(2*Df))
PC aa C28:1	1.25	1	1.12
Age	1.14	1	1.07
Sex	1.54	1	1.24
BMI	1.18	1	1.09
Smoking	1.38	2	1.08
Physical activity	1.07	1	1.03
HDL-C	1.80	1	1.34
Systolic Blood Pressure	1.12	1	1.06
Triglycerides	1.43	1	1.19
Fasting serum glucose	1.10	1	1.05

Figure S15: The linearity graphs for representative metabolite PC ae C30:0 for acylalkylphosphatidylcholine

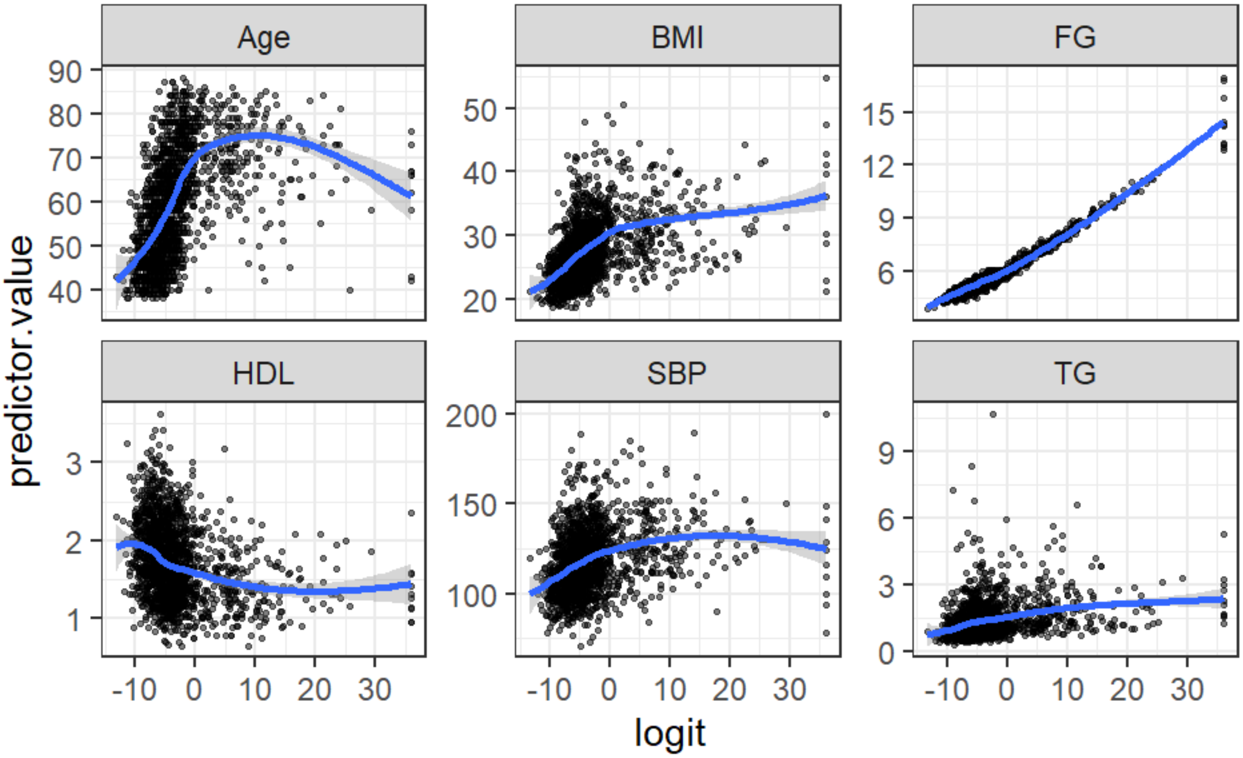

Table S27: VIF values for multicollinearity between predictors in the model for acylalkylphosphatidylcholine - PC ae C30:0

Predictors	GVIF	Df	GVIF^(1/(2*Df))
PC ae C30:0	1.18	1	1.09
Age	1.16	1	1.08
Sex	1.48	1	1.22
BMI	1.19	1	1.09
Smoking	1.42	2	1.09
Physical activity	1.07	1	1.03
HDL-C	1.74	1	1.32
Systolic Blood Pressure	1.12	1	1.06
Triglycerides	1.39	1	1.18
Fasting serum glucose	1.11	1	1.05

Figure S16: The linearity graphs for representative metabolite SM (OH) C14:1 for sphingomyelins

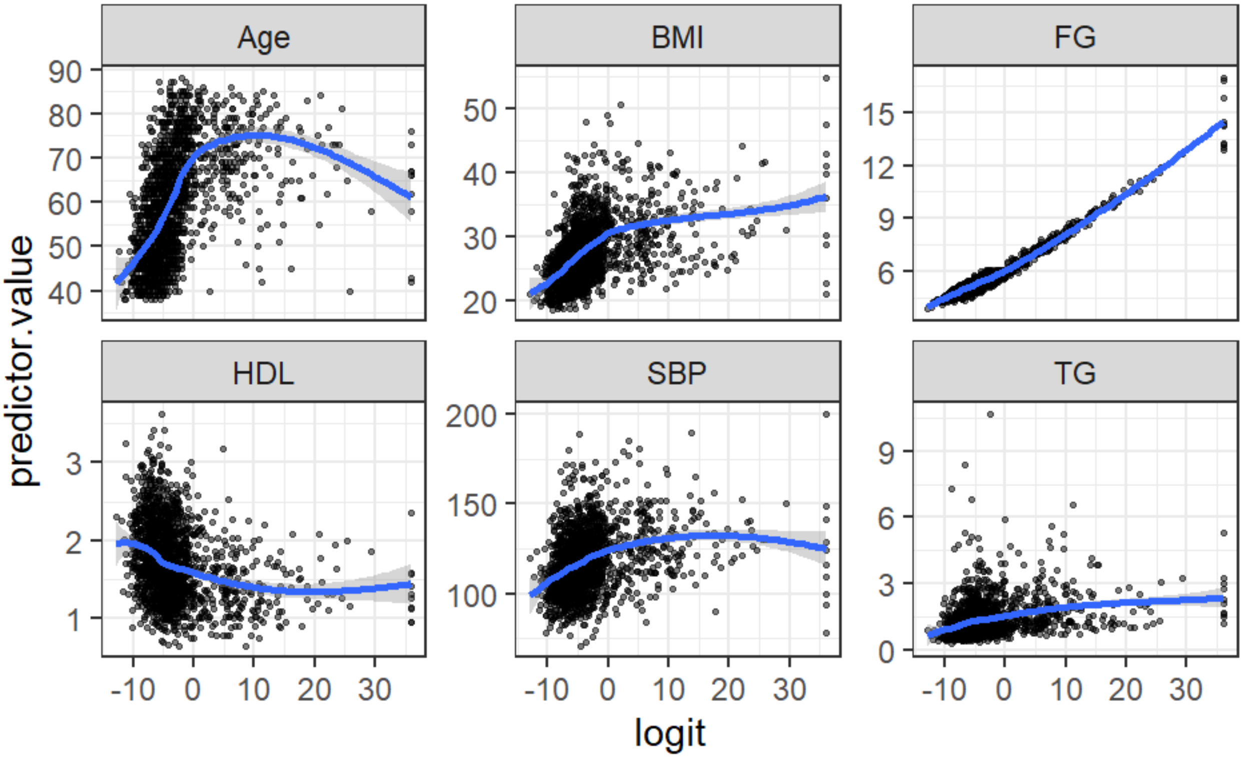

Table S28: VIF values for multicollinearity between predictors in the model for sphingomyelins - SM (OH) C14:1

Predictors	GVIF	Df	GVIF^(1/(2*Df))
SM (OH) C14:1	1.28	1	1.13
Age	1.20	1	1.10
Sex	1.47	1	1.21
Smoking	1.12	2	1.03
Physical activity	1.04	1	1.02
HDL-C	1.58	1	1.26
Systolic Blood Pressure	1.22	1	1.10
Triglycerides	1.33	1	1.15
Fasting serum glucose	1.25	1	1.12

Figure S17: The linearity graphs for representative metabolite Hexoses (H1)

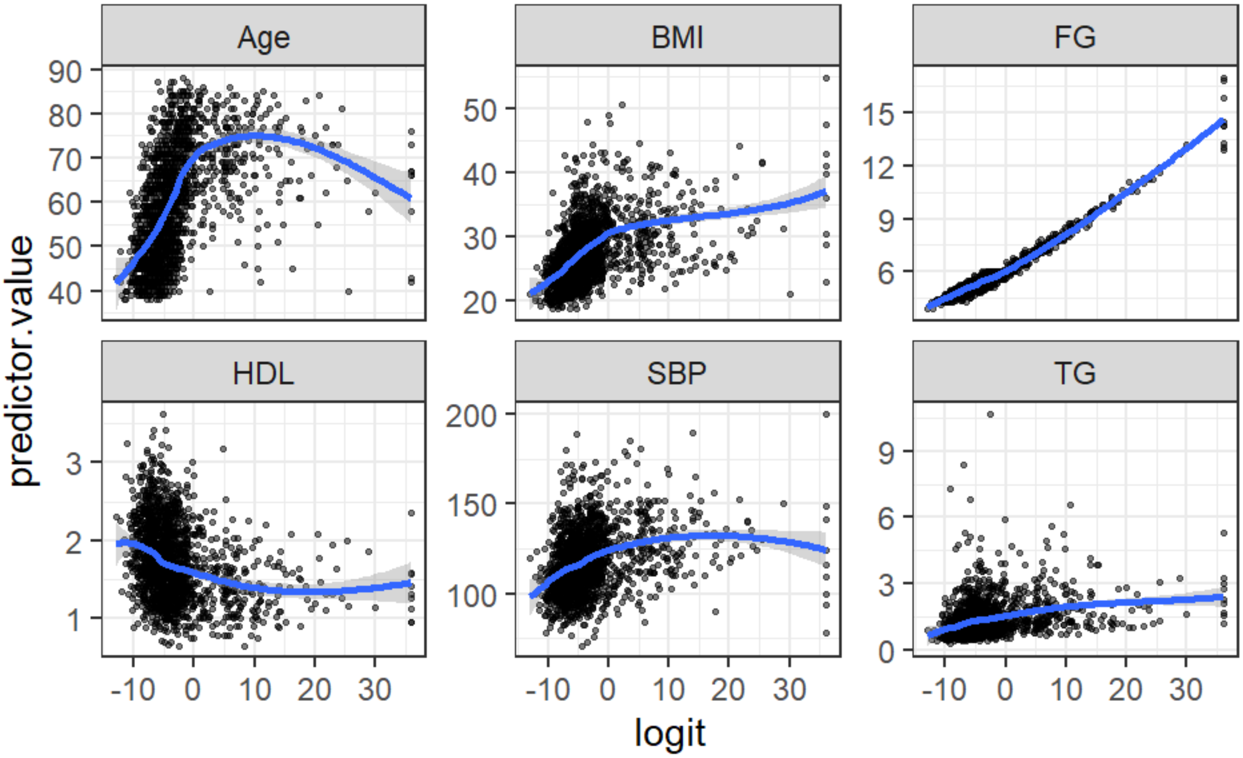

Table S29: VIF values for multicollinearity between predictors in the model for Hexoses (H1)

Predictors	GVIF	Df	GVIF^(1/(2*Df))
Hexoses	1.34	1	1.16
Age	1.14	1	1.07
Sex	1.47	1	1.21
BMI	1.18	1	1.09
Smoking	1.37	2	1.08
Physical activity	1.07	1	1.03
HDL-C	1.71	1	1.31
Systolic Blood Pressure	1.12	1	1.06
Triglycerides	1.38	1	1.17
Fasting serum glucose	1.40	1	1.18

Figure S18: The linearity graphs for representative metabolite alanine (Ala) of amino acids

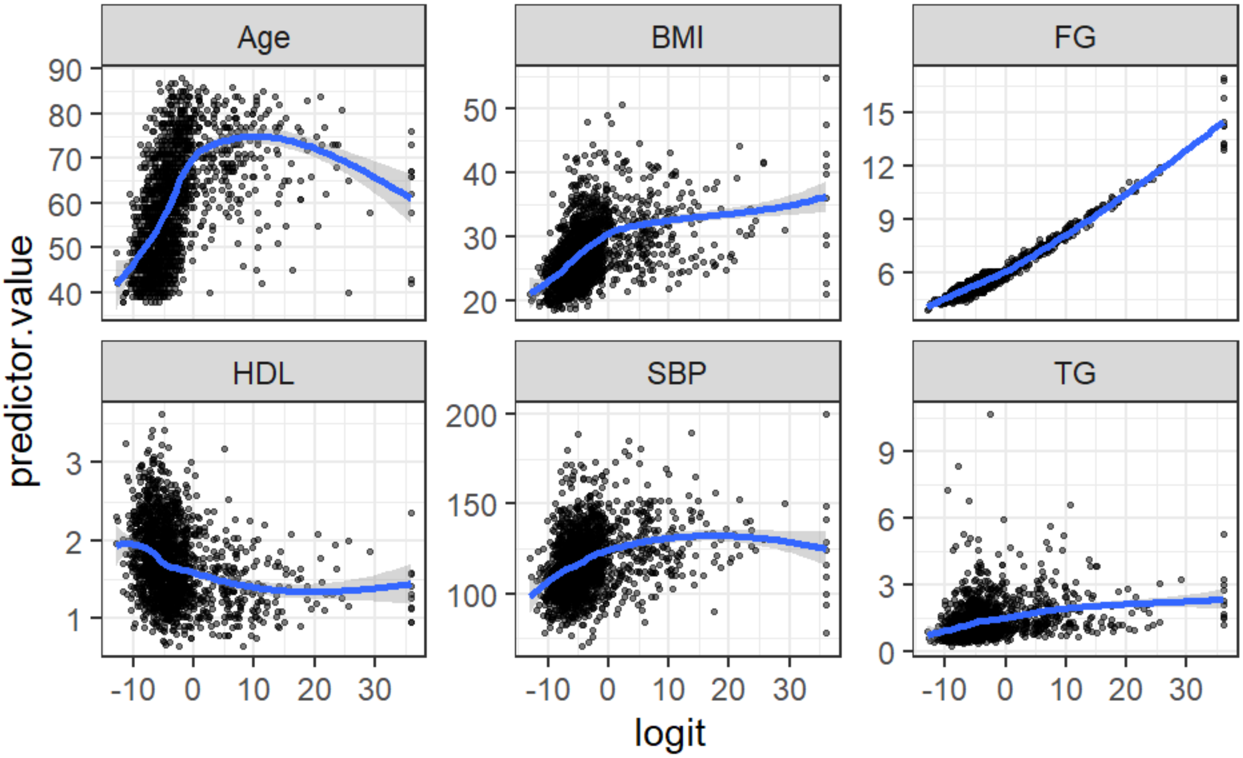

Table S30: VIF values for multicollinearity between predictors in the model for amino acids - alanine (Ala)

Predictors	GVIF	Df	GVIF^(1/(2*Df))
Alanine	1.11	1	1.05
Age	1.15	1	1.07
Sex	1.47	1	1.21
BMI	1.18	1	1.09
Smoking	1.39	2	1.09
Physical activity	1.07	1	1.03
HDL-C	1.70	1	1.31
Systolic Blood Pressure	1.12	1	1.06
Triglycerides	1.43	1	1.20
Fasting serum glucose	1.10	1	1.05

Figure S19: The linearity graphs for representative metabolite acetylornithine (Ac-Orn) of biogenic amines

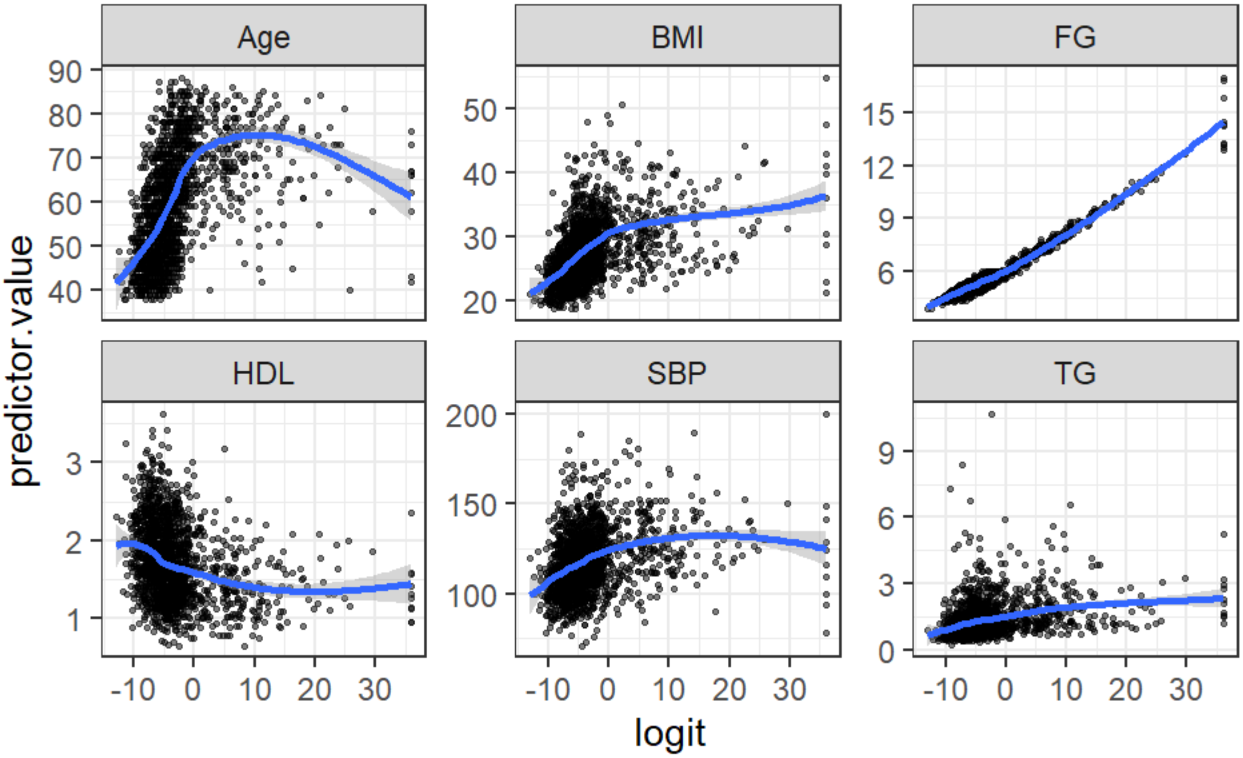

Table S31: VIF values for multicollinearity between predictors in the model for biogenic amines - acetylornithine

Predictors	GVIF	Df	GVIF^(1/(2*Df))
Acetylornithine	1.08	1	1.04
Age	1.16	1	1.08
Sex	1.49	1	1.22
BMI	1.18	1	1.09
Smoking	1.37	2	1.08
Physical activity	1.07	1	1.03
HDL-C	1.70	1	1.30
Systolic Blood Pressure	1.13	1	1.06
Triglycerides	1.39	1	1.18
Fasting serum glucose	1.10	1	1.05

Reference

1. James, G.; James, G.; Witten, D.; Hastie, T.; Tibshirani, R.; Ebscohost. *An introduction to statistical learning: with applications in R*, Uncorrected edition. ed.; Springer: New York, 2013.
